# *Plasmodium berghei* rhoptry neck protein 6 maintains parasite infectivity and virulence

**DOI:** 10.1128/mbio.01941-25

**Published:** 2025-09-25

**Authors:** Veeda Narahari, Smita Patri, Minal Dilip Satpute, Geeta Kashyap Vemuganti, Dilip Kumar Mishra, Surendra Kumar Kolli, Kota Arun Kumar

**Affiliations:** 1 Department of Animal Biology, School of Life Sciences, University of Hyderabad 28614, Hyderabad, Telangana, India; 2 School of Medical Sciences, University of Hyderabad 28614, Hyderabad, Telangana, India; 3 L V Prasad Eye Institute, Hyderabad, Telangana, India; Rutgers-New Jersey Medical School, Newark, New Jersey, USA

**Keywords:** *Plasmodium*, rhoptries, sporozoites, EEFs, hepatocytes, splenomegaly

## Abstract

Apicomplexan parasites possess unique secretory organelles called rhoptries, which are reservoirs for rhoptry neck proteins (RONs) and rhoptry bulb proteins (ROPs) that aid in host cell attachment, invasion, and intracellular proliferation. In *Plasmodium falciparum*, the RON6 (*Pf*RON6) locus is indispensable, limiting its functional investigation. Using a rodent malaria model, we show the role of *P. berghei* RON6 (PbRON6), a putative ortholog of *Pf*RON6, in the invasion of RBC, hepatocytes, and during liver stage development. *Pb*RON6 localizes to the sporozoite membrane and has an extracellular C-terminal domain. RON6-depleted parasites fail to maintain infectivity and virulence, leading to prolonged survival of mice. The mutants induce chronic malaria and hyper-reactive malarial splenomegaly, characterized by decreased B and T lymphocytes concurring with loss of lymphoid follicles. Our findings provide a rationale for targeting *Pb*RON6 in pre-erythrocytic stages to prevent clinical malaria and also for understanding the basis of hyper-reactive splenomegaly in mice using the *PbΔron6* mutant model.

**IMPORTANCE:**

*Plasmodium* sporozoites are infective to mammalian hepatocytes. Prior to entry into the cell, sporozoites release proteins from their apical cell organelles called micronemes and rhoptries. The secreted proteins contact the hepatocyte membrane to create a structure called a moving junction (MJ) that progressively invaginates inside the cell, utilizing the parasite’s actomyosin molecular motor. This activity finally culminates in establishing an intracellular vacuole that harbors the parasite. As MJ is crucial for intracellular infection, targeting the components of this complex has implications in reducing malaria infection. We show that a rhoptry resident protein, RON6, is required for the invasion of merozoites and sporozoites, together with a role in the development of parasites in the hepatocytes. Consistent with its probable role in hepatocyte invasion, the RON6 is localized to the sporozoite membrane, with its C-terminal domain being extracellular. Our observations reveal that RON6 maintains the virulence of the parasite, and mutants lacking RON6 enhance host survival and induce hyper-reactive malarial splenomegaly.

## INTRODUCTION

Malaria is caused by a unicellular apicomplexan parasite *Plasmodium* that is digenetic in nature, requiring two hosts to complete its life cycle. The disease is highly prevalent in tropical and subtropical areas of the world, including parts of South America, Asia, and Africa. Malaria kills nearly half a million people each year, with the majority of cases occurring in Sub-Saharan Africa. According to the WHO report of 2023, in 2022 alone, nearly 249 million people got infected with malaria, and approximately 0.6 million died due to the disease (1).

The malaria infection in the vertebrate host begins with the inoculation of sporozoites by the bite of a female *Anopheles* mosquito (2). The sporozoites quickly travel to the liver via circulation, invade hepatocytes, and develop into exoerythrocytic forms (EEFs) (3). Within the EEF, thousands of first-generation merozoites are formed as a result of the asexual schizogony (4). At the end of the EEF development, the hepatic merozoites are packaged into merosomes, specialized sac-like structures derived from hepatocyte plasma membrane and parasite proteins (5). These merosomes, upon rupture, initiate the erythrocytic cycle. Following repeated asexual cycles, some of the erythrocytic stages transform into sexually dimorphic forms called gametocytes that are infective to female *Anopheles* mosquitoes. Sexual reproduction occurs in the mosquito gut and results in the formation of a zygote, which transforms into a motile ookinete. Ookinetes breach the midgut epithelium and develop into an oocyst on the hemocoel side of the epithelium. Sporulation within the oocyst produces thousands of sporozoites (6), which are released into the hemocoel upon rupture. The sporozoites migrate and colonize the salivary glands (SGs) and wait for the next round of transmission to a new vertebrate host during blood-feeding.

Host cell invasion at multiple life cycle stages is key for malaria transmission, thus making this process an attractive target to control the disease. All apicomplexan parasites, including *Plasmodium* sporozoites, deploy three secretory organelles, viz., the micronemes, dense granules, and rhoptries for the secretion of a variety of proteins that aid in parasite motility, cell traversal, and host cell invasion (7). Post-invasion, some secretory proteins also contribute to the formation of parasitophorous vacuolar membrane (PVM) (8), a unique structure that prevents the endocytic fusion and cellular degradation of the intracellular parasites (9).

The rhoptries in *Plasmodium* schizonts are the largest, club-shaped secretory organelles (10) originating from the fusion of coated vesicles of the Golgi (11). They are delimited by a membrane and organized into a distinct neck and bulb region (11, 12), which respectively make up nearly 2% and 7% volume of the merozoite (12). As these are required for invasive stages, they arise *de novo* during sporogony and schizogony stages (13, 14), with the exception of the midgut invasive ookinete stage, where they are completely absent (15). Recent experimental approaches have identified nearly 45 and 11 proteins localizing to the rhoptries of merozoites and sporozoites, respectively (16). The rhoptry proteins perform a myriad of functions in asexual stages. In *Plasmodium falciparum*, there is a distinct division of labor for the activities of the neck and bulb proteins*,* with functions respectively in host cell contact, invasion, and remodeling. For example, the reticulocyte binding proteins like Rh1, Rh2a, Rh2b, Rh4, and Rh5 (17–22) that bind to host cell receptors and facilitate merozoite invasion of RBCs localize to the rhoptry neck. Other rhoptry neck proteins like RON2, RON4, and RON5 are involved in tight junction formation, along with micronemal proteins AMA1 (13). RON3 interacts with RON2 and RON4 but not with AMA1 and hence not a part of moving junction (MJ) complex (23). Interestingly, few RONs are conserved in other Apicomplexan parasites like *Toxoplasma* and *Eimeria*, pointing to a common role in host cell invasion mechanisms (13). In contrast, the ROPs are unique to each species of Apicomplexa, likely conferring them with distinct host specificities (13). For example, in *Toxoplasma,* the ROPs interfere with host cell signaling (24) and abrogate host cell immunity (25, 26), favoring the establishment of chronic infections (27). However, in *Plasmodium*, ROPs are involved in the biogenesis of rhoptries (28, 29), invasion of host cells (29, 30), PV formation (31), and modification of host cells (32, 33).

More recently, RONs have been implicated in the sporozoite invasion of SGs and hepatocytes. Conditional depletion of RON2 (34) and RON11 (35) in sporozoites impaired SG invasion and reduced hepatocyte infectivity. A well-characterized protein complex of RON2, RON4, and RON5 implicated in merozoite invasion of RBC was also shown to be critical for sporozoite invasion of SGs (36). The study demonstrated a requirement of RON2 interaction with RON4 to form a stable complex that eventually facilitates sporozoite attachment to the substrate. An *in vivo* role of RON4 has also been demonstrated in gliding and infection of hepatocytes (37). Interestingly, while RON12 localizes to the rhoptry bulb in oocyst and SG sporozoites, it has no role in sporozoite invasion of SGs or hepatocytes, and its depletion does not affect EEF development (38).

The role of RON6 has not been studied in the sporozoite and liver stages of *Plasmodium* till date. In the current study, we provide a comprehensive analysis of the stage-specific localization of RON6 across all stages of *P. berghei*. Importantly, we show the localization of *Pb*RON6 on the sporozoite membrane, bearing an extracellular domain and also its association with PVM in liver stages. The study also identified novel interacting partners of *Pb*RON6 in the merozoite stage. We also generated a *Pbron6* deletion mutant (*PbΔron6*) and showed its dispensable nature across all life cycle stages. Nonetheless, the invasive stages of mutant merozoites and sporozoites revealed a dramatic decrease in infectivity to host cells. Interestingly, the *PbΔron6* mutants manifested compromised asexual propagation and erythrocytic reinvasion, reduction in hepatic growth and schizogony and delayed pre-patency, collectively leading to reduced parasite virulence. Concomitant with this observation, the mutants induced a condition of hyper-reactive malarial splenomegaly. Immunophenotyping of splenocytes from mutant-infected mice revealed reduced B and T lymphocytes, pointing to an altered immunological niche in the spleen.

## RESULTS

### Bioinformatics analysis revealed *Pb*RON6 is extracellular

**Fig 1:**
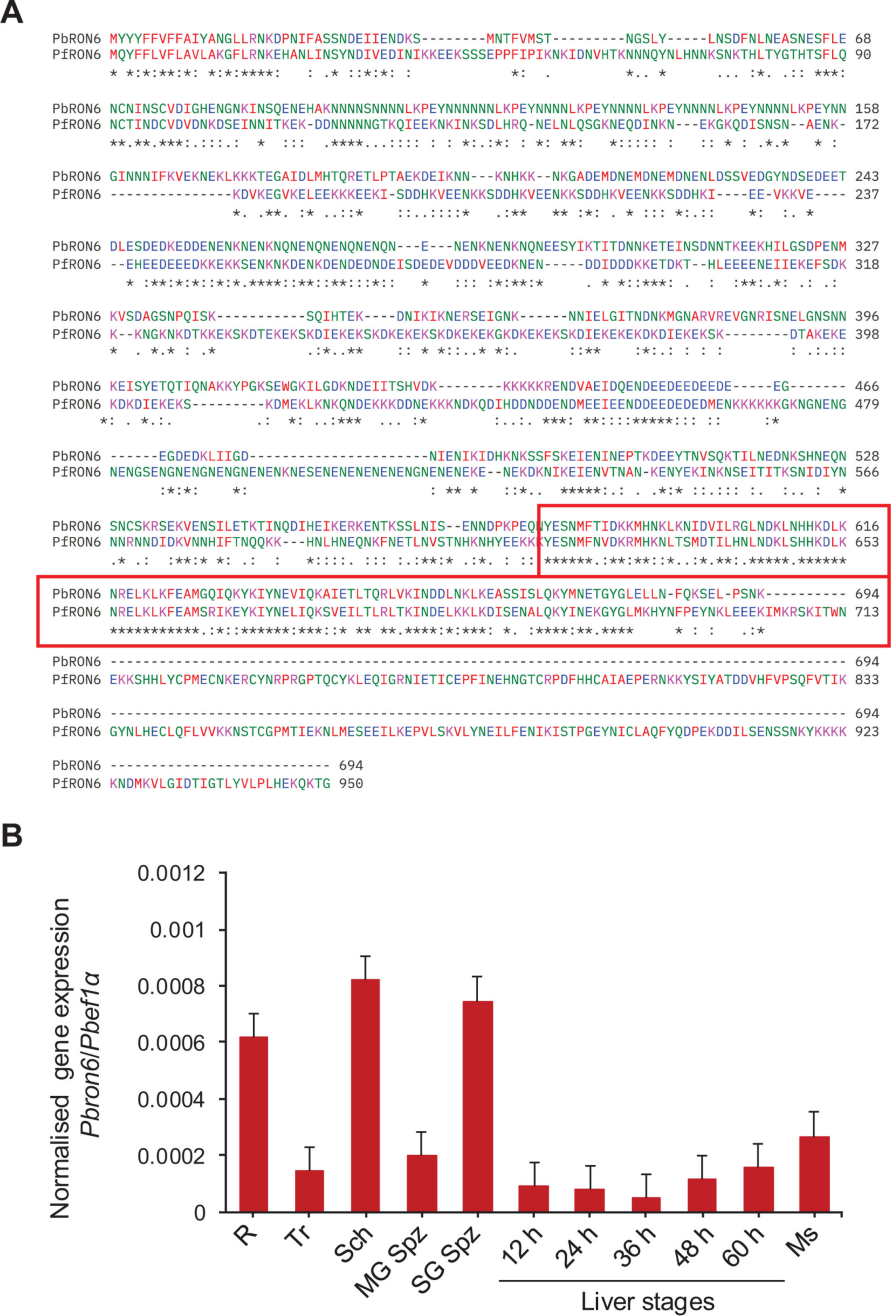
Sequence alignment of *Pb*RON6 and *Pf*RON6, and gene expression analysis of *Pbron6* (**A**) Amino acid sequence alignment of *P. berghei* RON6 (*Pb*RON6) and *P. falciparum* RON6 (*Pf*RON6). Asterisk (*) indicates identical amino acid residues, colon (:) indicates the alignment has strongly similar amino acids and dot (.) indicates alignment has weakly similar amino acids. The sub-C-terminal conserved domain is shown in the red box. *Pf*RON6 has an additional 217 amino acid sequence at its C-terminus. (**B**) Gene expression analysis of *Pbron6* from different life cycle stages by quantitative real-time PCR. The cDNAs generated from different stages are indicated as—R: rings, Tr: trophozoites, Sch: schizonts, MG Spz: Midgut sporozoites, SG Spz: Salivary gland sporozoites, 12 h, 24 h, 36 h, 48 h, 65 h—time points of liver stages after sporozoite infection of HepG2 cells and Ms: Merosomes collected from the supernatants of liver stage cultures. Data normalization was performed by taking the ratio of absolute copy numbers of *Pbron6* and *Pbef1α*.

PlasmoDB annotated PBANKA_0311700 as putative rhoptry neck protein 6 in *P. berghei* (*Pb*RON6), and the gene ID of RON6 in *P. falciparum* is PF3D7_0214900 (*Pf*RON6). The predicted protein sequences of *Pb*RON6 and *Pf*RON6 had 694 and 950 amino acids (AAs), respectively (Fig. 1A). Functional domain analysis using the DTU/Deep TMHMM prediction tool inferred both *Pf*RON6 and *Pb*RON6 to be globular proteins with no transmembrane domains. A signal sequence was predicted in both *Pb*RON6 and *Pf*RON6 from 1 to 15 AAs, and the sequence beyond 15 AAs was predicted as an extracellular domain (Fig. S1A and B). Signal P-4.1 tool was used to validate the DTU/Deep TMHMM predictions that identified a signal sequence in *Pf*RON6 between AAs 1 and 15, but no signal sequence was detected in *Pb*RON6. The unprocessed cleavage site was represented as *C*-score, whereas the presence of a signal peptide site was indicated as *S*-score, and the combination of both *C*- and *S*-scores was represented as *Y*-score. A cut-off value greater than 0.45 indicated the presence of a signal peptide in the *Pf*RON6 (Fig. S1C and D). Phobious web server predicted *Pb*RON6 and *Pf*RON6 as non-cytosolic proteins (Fig. S1E and F). All the orthologs of RON6 had a signal peptide and a conserved C-terminal region. An additional C-terminal cysteine-rich region was present in the RON6 orthologs of human and primate species (Fig. S1G).

### *Pbron6* transcript is highly expressed in schizonts and SG sporozoites

To investigate the expression profile of *ron6* across all the life cycle stages of *P. berghei*, we performed quantitative real-time PCR (qRT-PCR) by the absolute quantification method. The gene expression of *Pbron6* was normalized by taking the ratio of *Pbron6* and *Pbef1α* copy numbers. Normalized data showed maximal expression of *Pbron6* in the schizonts and SG sporozoites, followed by the ring stage, while other stages showed detectable levels of expression (Fig. 1B).

### *Pb*RON6 is expressed in asexual blood stages, sporozoites, and liver stages

**Fig 2:**
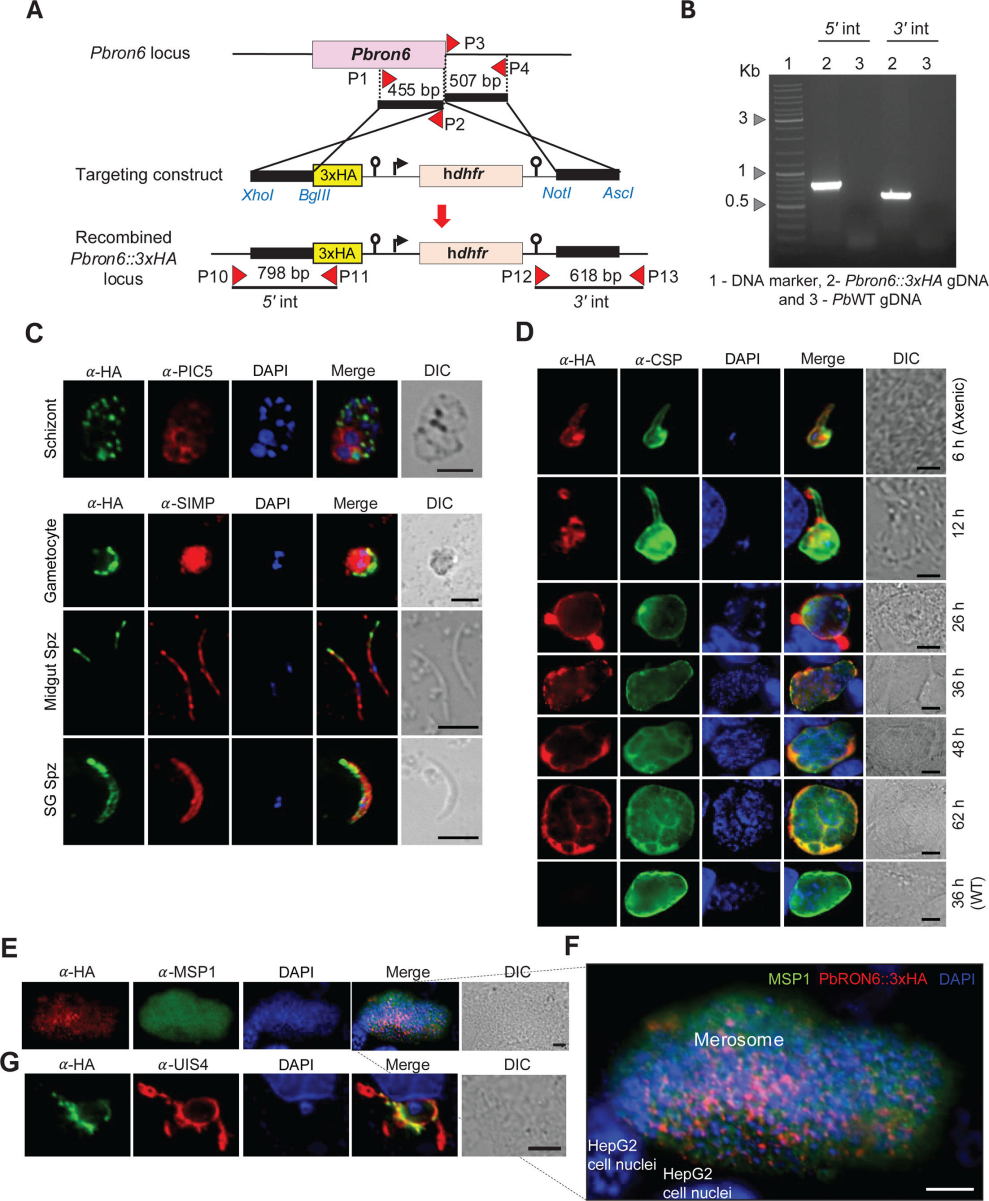
Generation of *Pbron6::3xHA* tagged line and analysis of HA expression across different life cycle stages. (**A**) Schematic showing the strategy for tagging 3xHA in frame with *Pbron6 orf*. The top panel shows the genomic locus of *Pbron6*. The middle panel shows the engineered *Pbron6::3xHA* targeting construct carrying h*dhfr* selectable marker cassette flanked by *Pbron6 orf* and *3′ utr* to facilitate double crossover homologous recombination. The solid black lines indicate regions selected for homologous recombination. The lower panel shows *Pbron6::3xHA* recombined locus. Red triangles indicate the primer positions, and the black line indicates the expected PCR product size to confirm the integrations by diagnostic PCRs. (**B**) Agarose gel images showing amplicons of diagnostic PCRs from *Pbron6::3xHA* genomic DNA (gDNA) confirming correct *5*′ and *3*′ integrations. (**C**) Analysis of *Pb*RON6::3xHA expression in schizont, gametocyte, midgut, and salivary gland sporozoites (Spz) by indirect immunofluorescence assay (IFA). Each stage (as labeled) was stained with anti-HA rabbit monoclonal antibody and stage-specific markers, as indicated. Immunoreactivity was revealed respectively with Alexa Fluor 488-conjugated anti-rabbit and Alexa Fluor 594-conjugated anti-mouse secondary antibodies. Nuclei were stained with DAPI. Scale bar: 5 µm. (**D**) Analysis of *Pb*RON6::3xHA expression in developmental stages of EEFs. Axenic and intrahepatic EEFs at indicated time points were stained with anti-HA rabbit monoclonal antibody and anti-CSP (3D11) mouse monoclonal antibody. The immunoreactivity was revealed respectively with Alexa Fluor 594-conjugated anti-rabbit and Alexa Fluor 488-conjugated anti-mouse secondary antibodies. The last panel shows a 36-h WT EEF stained with the same combination of antibodies, confirming the specificity of HA signals in transgenic parasites. Nuclei were stained with DAPI. Scale bar: 10 µm. (**E**) Merosomes obtained from 65 h EEF cultures were fixed and stained with anti-HA rabbit monoclonal and anti-MSP1 mouse monoclonal antibody. Immunoreactivity was revealed respectively with Alexa Fluor 594-conjugated anti-rabbit and Alexa Fluor 488-conjugated anti-mouse secondary antibodies. Nuclei were stained with DAPI. Scale bar: 10 µm. (**F**) Enlarged image of merosome with individual merozoites showing RON6-HA immunoreactivity at the apical end. Scale bar: 10 µm. (**G**) Colocalization of *Pb*RON6::3xHA with PVM marker UIS4. EEFs at 36 h were fixed and stained with anti-HA mouse monoclonal and anti-UIS4 (rabbit) primary antibody. Immunoreactivity was revealed with Alexa Fluor 488-conjugated anti-mouse and Alexa Fluor 594-conjugated anti-rabbit secondary antibodies. Nuclei were stained with DAPI. Scale bar: 10 µm.

To study the stage-specific protein expression and cellular localization of *Pb*RON6 throughout the parasite life cycle, a 3xHA (hemagglutinin) tag was translationally fused to *Pbron6 orf* at the C-terminus, excluding the stop codon, using standard genetic modification methods (Fig. 2A; Fig. S2A and B). Site-specific integration of the 3xHA localization construct in *Pbron6::3xHA* parasite genome was confirmed by integration-specific diagnostic PCRs (Fig. 2B). The presence of 3xHA in frame with *Pbron6* was confirmed by PCR and Sanger sequencing (Fig. S2C). Indirect immunofluorescence assay (IFA) across the life cycle stages showed punctate staining of HA in the schizont stage, likely suggestive of a rhoptry compartmentalization of *Pb*RON6 in individual merozoites. Gametocytes showed discrete patches of HA expression towards the periphery. Midgut sporozoites showed immunoreactivity in the apical region, indicative of a rhoptry localization of *Pb*RON6, whereas SG sporozoites showed HA expression both in the rhoptries and on the sporozoite membrane. *P. berghei* PIC5 was used as a marker for schizonts, and SIMP (39) was used as a marker for gametocytes, midgut, and SG sporozoites (Fig. 2C). Next, we investigated the subcellular distribution of *Pb*RON6 in the hepatic stages. We noted HA expression in the 6 h axenic (cell-free) cultures that continued in other stages of EEF development, corresponding to 12, 26, 36, 48, and 62 h in HepG2 cells. Interestingly, at 26 h and other later stages of EEF development, HA staining was observed on the EEF periphery, reminiscent of the staining pattern of PVM (Fig. 2D). We further investigated the 65 h EEFs in HepG2 cells, a point when hepatic merosomes are generated, and HA immunoreactivity was observed in individual hepatic merozoites, which points to the rhoptry localization of *Pb*RON6::3xHA (Fig. 2E and F). Taken together, sporozoites and liver stages showed high expression of *Pb*RON6::3XHA, which may hint at a likely role in maintaining the infectivity in both stages.

### *Pb*RON6 colocalized with PVM marker UIS4 in liver stages

During parasite invasion, the proteins of the rhoptry compartment are utilized for two purposes viz., for making a MJ, and second to facilitate the formation of PVM (8). To reiterate our previous observation of *Pb*RON6::3xHA association within the periphery of EEFs, we stained 36 h *in vitro* liver stages, with HA antibody and UIS4 antibody, a marker for PVM. At multiple foci, *Pb*RON6::3xHA was colocalized with UIS4 (Fig. 2G) and the Pearson correlation coefficient for colocalization was close to 0.8 (Fig. S3A). These studies confirmed that the peripheral HA signals covering the EEF throughout development (Fig. 2D) were indeed from PVM.

### The C-terminus of *Pb*RON6 is extracellular

**Fig 3:**
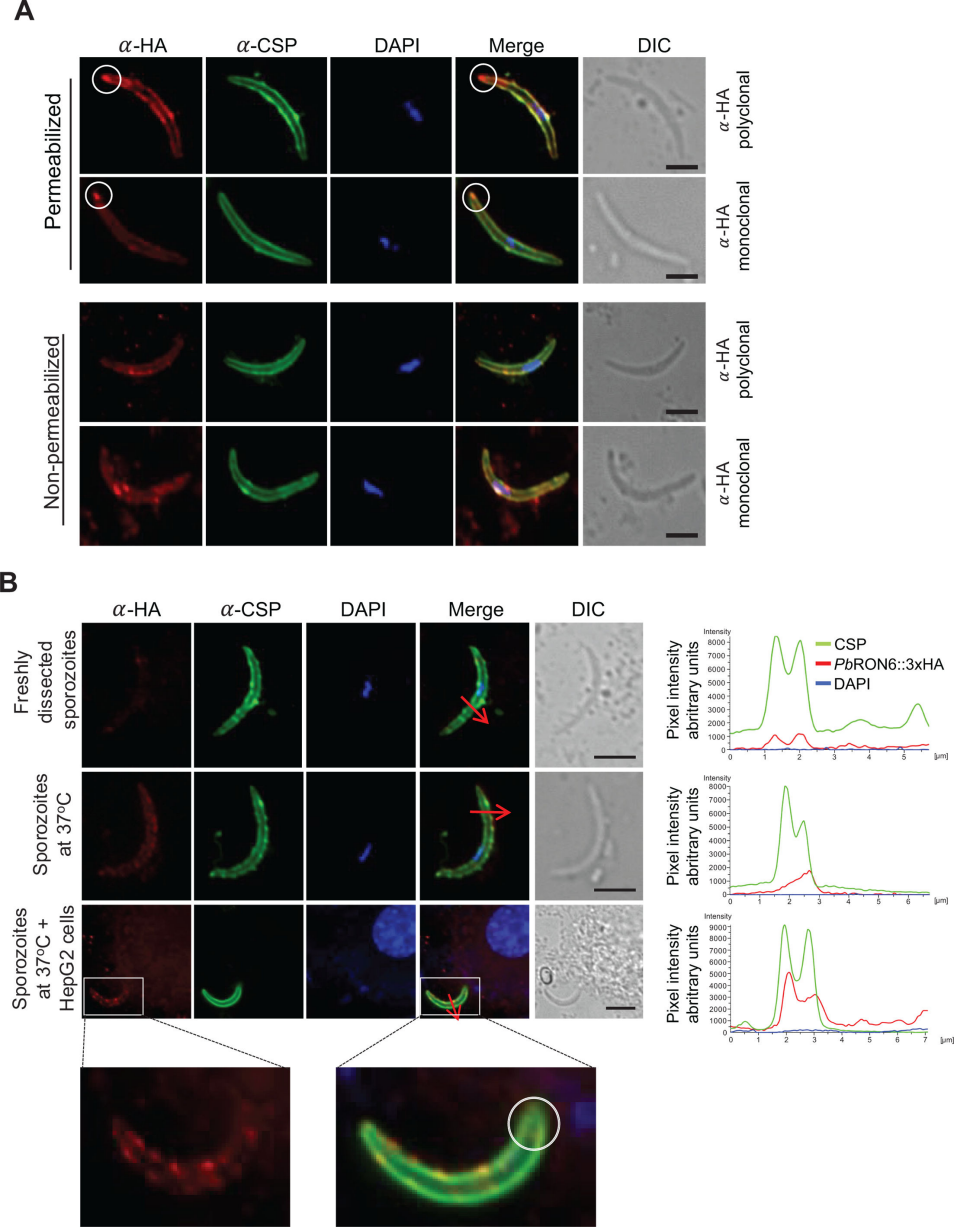
*Pb*RON6 is associated with the sporozoite membrane with an extracellular C-terminus. (**A**) Salivary gland sporozoites of *Pb*RON6::3xHA were fixed and immunostained with either anti-HA rabbit polyclonal or anti-HA rabbit monoclonal antibodies along with anti-CSP mouse monoclonal antibody under permeabilized (top panel) and non-permeabilized (bottom panel) conditions. Immunoreactivity was revealed, respectively, with Alexa Fluor 594-conjugated goat anti-rabbit and Alexa Fluor 488-conjugated chicken anti-mouse secondary antibodies. The apical localization of PbRON6::3xHA is shown in white circles (top panel). Nuclei were stained with DAPI. Scale bar: 5 µm. (**B**) The levels of *Pb*RON6::3xHA associated with the sporozoite membrane were assessed in three different conditions—immediately after isolation from salivary glands (top panel), after incubation of sporozoites at 37°C for 30 min (middle panel), and after incubation of sporozoites at 37°C for 30 min in the presence of HepG2 cells (bottom panel). The sporozoites were stained with anti-HA rabbit and anti-CSP mouse monoclonal antibodies. Immunoreactivity was revealed, respectively, with Alexa Fluor 594-conjugated goat anti-rabbit and Alexa Fluor 488-conjugated chicken anti-mouse secondary antibodies. Nuclei were stained with DAPI. An enlarged inset from the bottom panel shows the accumulation of *Pb*RON6::3xHA at discrete foci on the sporozoite membrane. The right panels show the pixel intensity profile and colocalization of HA and CSP for all three conditions measured by cross-sectional imaging using NIS-element AR software. Scale bar: 5 µm.

Our IFA studies showed an association of *Pb*RON6 with the apical end and with the membrane of SG sporozoites. The possible orientation of the C-terminal HA tag was tested by performing IFA with and without permeabilization. Sporozoites were stained with 3D11, which binds to a GPI-anchored CSP (40) and HA antibodies (Fig. 3A). The immunoreactivities of CSP and HA showed colocalization, providing compelling evidence that the membrane-associated *Pb*RON6 has an extracellular C-terminal domain. This observation was in agreement with the DTU/Deep TMHMM that predicted amino acids 16–694 to be extracellular.

### *Pb*RON6::3xHA appeared as discrete speckles on the sporozoite membrane following exposure to 37°C and HepG2 cells

The proteins of the rhoptry compartment have been implicated in both the formation of the MJ and PVM (8). For the formation of a MJ, the rhoptry protein accumulates at the apical end of the zoite and is secreted onto the host cell membrane, thereby establishing a connection with a preformed RON2-AMA1 complex (41). As these events are also conserved during the hepatic sporozoite invasion (36), we reasoned whether temperature shift and hepatocyte contact upregulated the sporozoite-specific expression of *Pb*RON6::3XHA. To this end, we analyzed *Pb*RON6::3xHA expression on sporozoites under three conditions, viz., immediately after isolation from SGs, 30 min after exposure to 37°C, and 30 min after exposure to 37°C in the presence of HepG2 cells. The *Pb*RON6::3XHA levels were analyzed by IFA using HA antibody, and the sporozoites were counter-stained with 3D11 (Fig. 3B). We noted very meager levels of *Pb*RON6::3xHA in immediately dissected sporozoites, while sporozoites exposed to 37°C had some appreciable levels of protein appearing on the sporozoite membrane. However, when incubated with host cells, we noted a dramatic accumulation of *Pb*RON6::3xHA at several foci, as discrete spots on the sporozoite membrane. We conclude that *Pb*RON6::3xHA accumulates on sporozoite membrane when exposed to 37°C and further, its levels increases in contact with hepatocytes. The levels of PbRON6::3xHA were confirmed by measuring fluorescence pixel intensity across the sporozoite membrane (Fig. 3B).

### *Pb*RON6::3xHA is not released during sporozoite exocytosis

**Fig 4:**
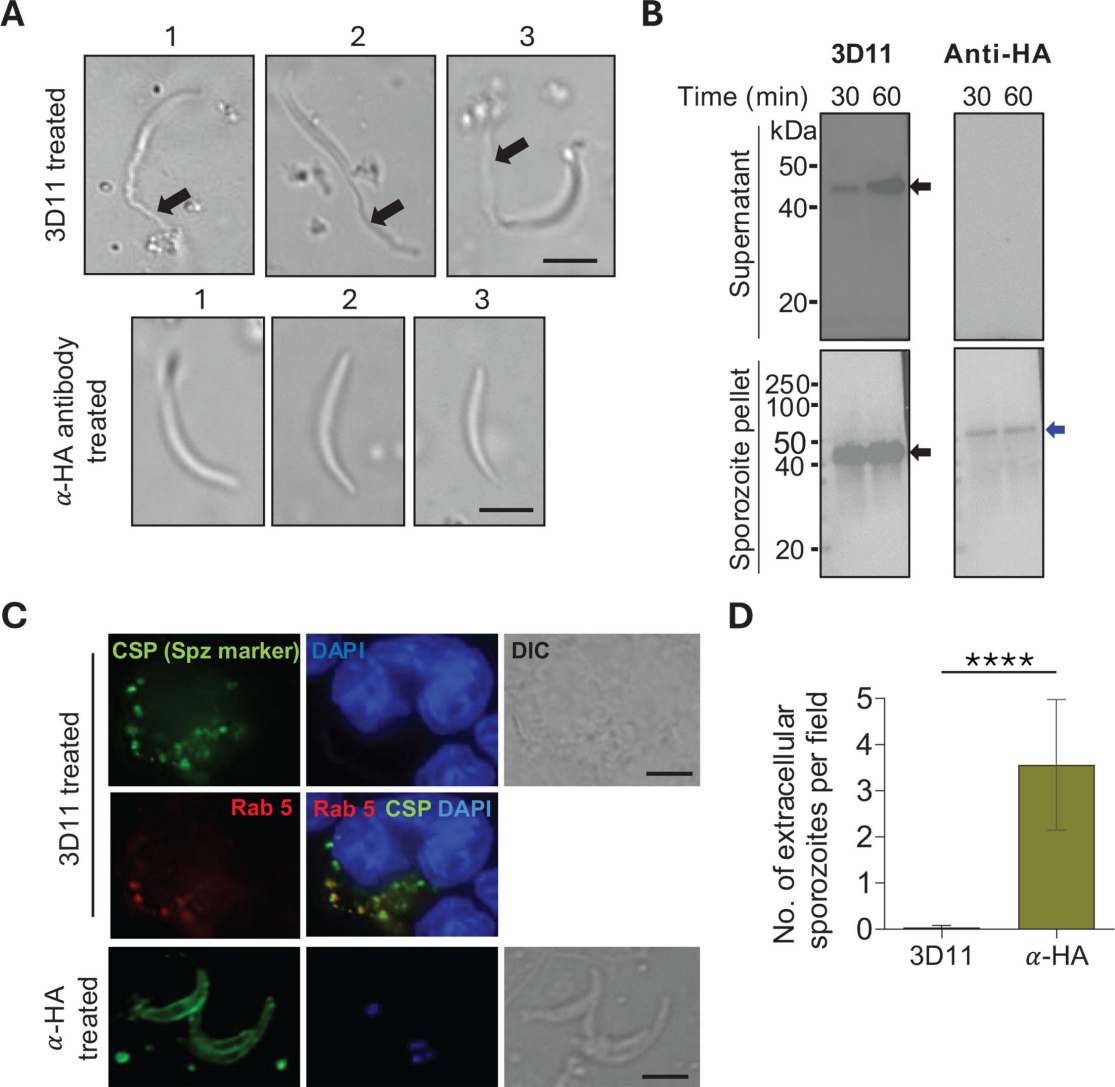
*Pb*RON6::3xHA is not released during sporozoite exocytosis. (**A**) Exocytosis of *Pb*RON6::3xHA sporozoites. Representative images of *Pb*RON6::3xHA sporozoites treated with 3D11 (top panel) and anti-HA rabbit monoclonal antibody (lower panel), in three independent experiments: 1, 2, and 3. Scale bar: 5 µm. (**B**) Immunoblots showing exocytosis of 5 × 10^4^ *Pb*RON6::3xHA transgenic sporozoites in RPMI medium with 10% FBS, incubated for 30 and 60 min at 37°C. Supernatant (top panel) and pellet (bottom panel) fractions were subjected to SDS-PAGE followed by western blot analysis. The membranes were probed with 3D11, which recognizes the central repeat region of CSP, and anti-HA rabbit monoclonal antibodies. The immunoreactivity was revealed with anti-mouse and anti-rabbit secondary antibodies conjugated with HRP, and the bands were visualized by the ECL detection. A time-dependent exocytosis was noted in the supernatant only for CSP (black arrows) but not for *Pb*RON6::3xHA. The sporozoite pellet showed immunoreactivity for both CSP (black arrow) and *Pb*RON6::3xHA (blue arrow). (**C**) Fate of 3D11 and anti-HA rabbit monoclonal treated *Pb*RON6::3xHA transgenic sporozoites in the mouse macrophage (RAW) cell line. *Pb*RON6::3xHA sporozoites were incubated with either 3D11 or anti-HA rabbit monoclonal antibodies for 30 min at room temperature and added to the RAW cell line. After 7 h, indirect immunofluorescence assay was performed using 3D11 to visualize sporozoites and rabbit anti-Rab5 was used to stain macrophage endosomes. The immunoreactivity was revealed with Alexa Fluor 488-conjugated anti-mouse and Alexa Fluor 594-conjugated anti-rabbit secondary antibodies. Nuclei were stained with DAPI. Scale bar: 5 µm. (**D**) Bar graph showing quantification of extracellular sporozoites treated with 3D11 or anti-HA. Error bars represent mean with standard deviation (****P < 0.0001, Mann-Whitney test).

As our studies showed an extracellular nature of the C-terminal domain of *Pb*RON6, we next investigated the possible inactivation of *Pb*RON6::3xHA tagged sporozoites, following their exposure to anti-HA antibodies. Sporozoite inactivation following exposure to 3D11 is well known owing to the binding of the antibody to CSP repeats present on the sporozoite membrane. The CSP-antibody complex is shed from the posterior end of the sporozoite in a characteristic “precipitin reaction” that can be visualized under a microscope. This complex gets sloughed off during sporozoite gliding movement (42), finally leading to their immobilization (43) (Fig. 4A). The shedding of the CSP-antibody complex is due to the exocytosis of CSP, following its proteolytic cleavage by a sporozoite membrane resident cysteine protease (44). Since we demonstrated that *Pb*RON6 is localized on the sporozoite membrane, we asked if it is also secreted through exocytosis. In three independent experiments, we incubated 5 × 10^3^ *Pb*RON6::3xHA sporozoites with 1 µg of anti-HA rabbit monoclonal or 3D11 at 37°C for 30 min and visualized under a microscope. As expected, the sporozoites treated with 3D11 exhibited precipitin reaction; however, no such activity was noted with the anti-HA antibodies (Fig. 4A). In fact, anti-HA antibody treatment did not arrest the motility of the *Pb*RON6::3xHA transgenic sporozoites. To rule out the possibility of low or undetectable levels of *Pb*RON6 released during exocytosis, we analyzed the levels of *Pb*RON6::3xHA in media incubated with sporozoites for 30 and 60 min. The supernatant and pellet fractions were analyzed by immunoblotting to check the levels of CSP and *Pb*RON6::3xHA (Fig. 4B). We noted a dose-dependent release of CSP in the supernatants, while *Pb*RON6::3xHA was not released. However, in the pellet fraction, we noted *Pb*RON6::3xHA expression, whose levels were much lower compared to CSP. We conclude that no detectable processing of *Pb*RON6 likely happens on the surface of the sporozoite.

### Anti-HA antibody-treated *Pb*RON6::3xHA sporozoites are not opsonized

Opsonization is the most common outcome of antibody coating the pathogens and is mediated by a variety of Fc and complement receptors present on phagocytic cells (45, 46). We analyzed whether anti-HA treated sporozoites were subjected to phagocytosis *in vitro*. As a control, we treated sporozoites with 3D11. Both groups of treated sporozoites were added to the RAW, a mouse macrophage cell line. We noted that the majority of 3D11-coated sporozoites were taken up by the RAW cells as observed at 7 h post-addition (Fig. 4C, upper panel and Fig. 4D). These sporozoites were predominantly intracellular and appeared disintegrated as revealed by 3D11 staining. Interestingly, the CSP staining colocalized with Rab5, an early endosomal marker (Fig. 4C, middle panel). However, the anti-HA antibody-treated *Pb*RON6::3xHA sporozoites were predominantly extracellular, indicating their lack of opsonization (Fig. 4C, lower panel and Fig. 4D).

### *Pb*RON6 interactome majorly included clients involved in host cell invasion and remodeling

**Fig 5:**
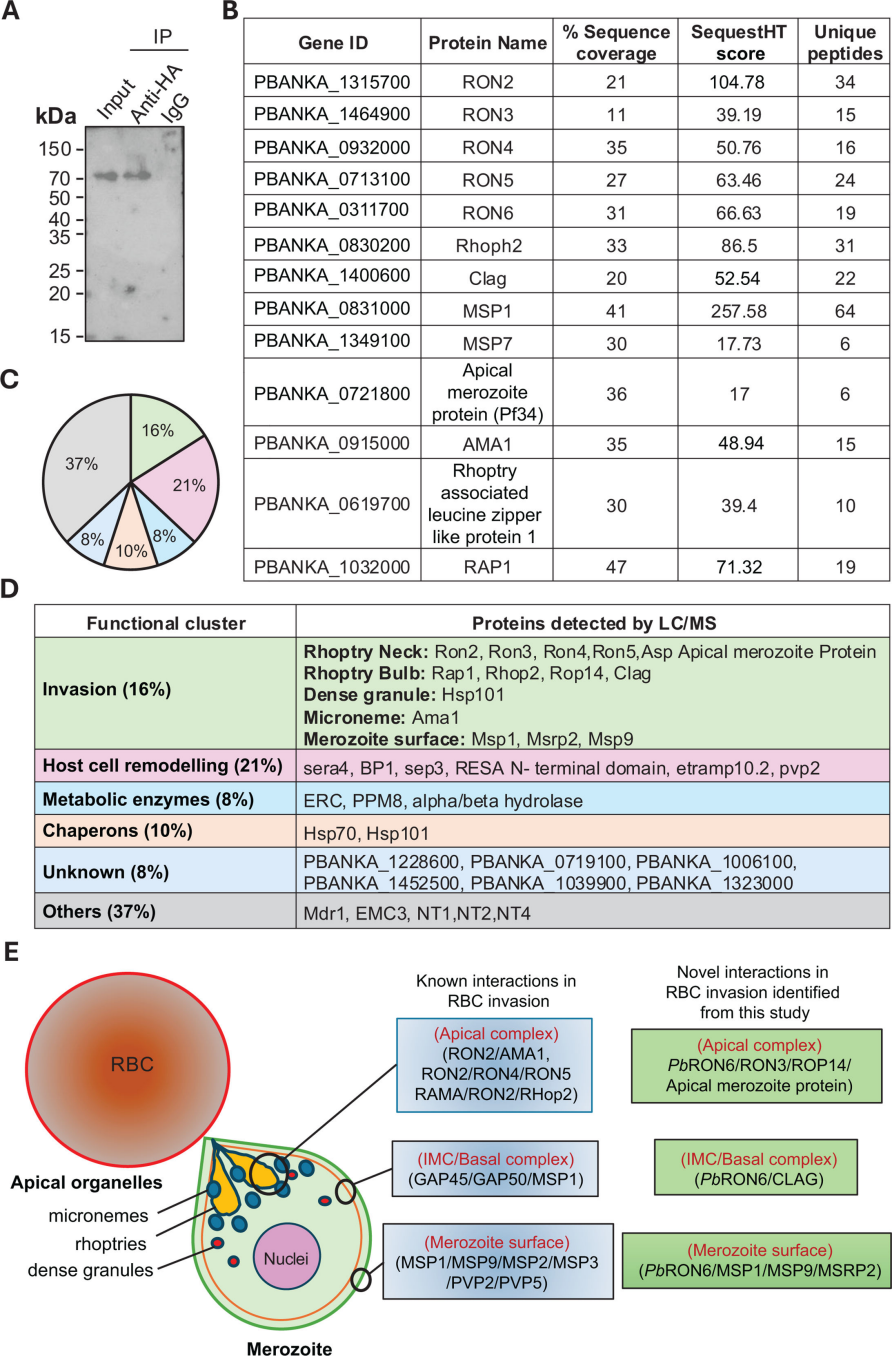
Identification of *Pb*RON6 interactome by LC/MS analysis. (**A**) Western blot showing the presence of *Pb*RON6 both in the schizont lysate (input) and proteins pulled down using anti-HA antibody. *Pb*RON6 was not detected in immunoprecipitation (IP) performed with pre-immune IgG (negative control). (**B**) List of *Pb*RON6 interacting partners identified by LC/MS analysis with significant sequestHT score. (**C**) Pie diagram showing the percentage of identified proteins categorized in individual clusters identified by functional gene ontology (GO). (**D**) Functional clusters showing the list of proteins interacting with RON6 detected in LC/MS analysis by co-IP using anti-HA antibody. (**E**) Schematic showing the probable novel invasion-related complex identified in this study.

To investigate the interacting partners of *Pb*RON6, we performed co-immunoprecipitation (IP) followed by LC/MS-based protein identification. The HA antibody or pre-immune IgG was conjugated to protein A/G beads and incubated with schizont lysates prepared from *Pb*RON6::3xHA line. The elute was analyzed on an immunoblot, which revealed the presence of *Pb*RON6::3xHA protein only in IP with HA antibody and not in IgG control (Fig. 5A). The most probable interacting partners of PbRON6 for which more than two peptides were detected in LC/MS analysis are shown in Fig. 5B.

The interactome study identified proteins involved in merozoite invasion, like apical complex proteins such as RON2, RON3, ROP14, and apical merozoite protein. The inner membrane complex protein CLAG and the merozoite surface proteins MSP1, MSP9, and MSRP2 were also identified (Fig. 5B). Relative percentage of protein clusters interacting with *Pb*RON6 was indicated as invasion related (16%), metabolic enzymes (8%), chaperones (10%), others (37%), and unknown (8%) (Fig. 5C). A few representative candidates within each cluster are shown in Fig. 5D. Each cluster was assigned a GO term ID based on the fold enrichment (*P* value < 0.01) (Fig. S5). A model was generated to show known parasite proteins interacting with RBC and those identified in the current study (Fig. 5E).

### The *PbΔron6* parasites exhibited slow asexual growth and decreased virulence

**Fig 6:**
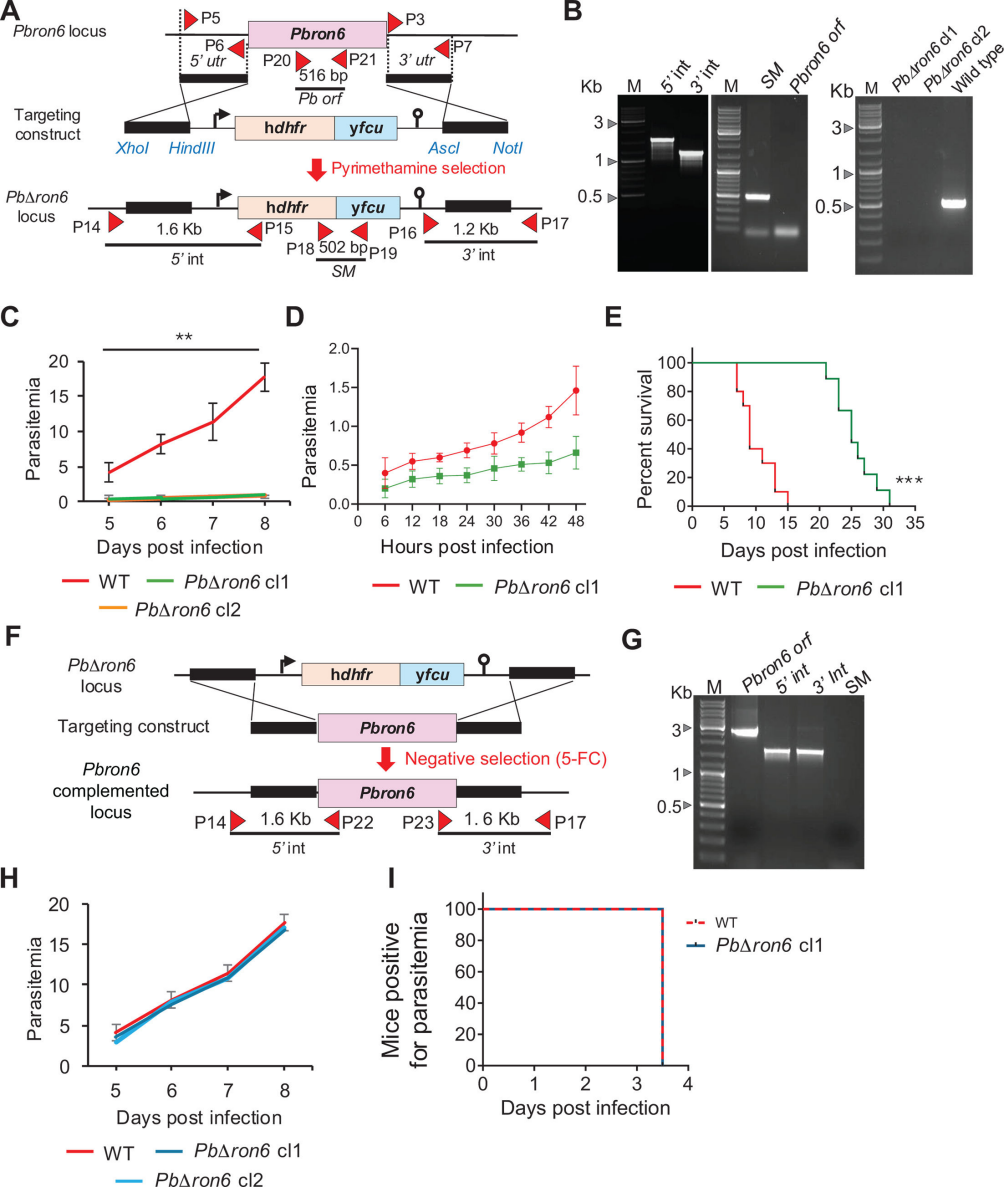
*Pbron6* is required for normal asexual propagation. (**A**) Strategy to generate *PbΔron6* by double crossover homologous recombination. The top panel shows the genomic locus of *Pbron6*. The middle panel shows *Pbron6* knockout targeting construct carrying h*dhfr* and y*fcu* selectable marker (SM) cassette, flanked by *Pbron6 5*′ and *3′ utr* regions for double crossover homologous recombination. The solid black lines indicate regions selected for homologous recombination. The lower panel shows the recombined locus, where the *Pbron6 open reading frame* (*orf*) is replaced with a SM cassette. Red triangles indicate the primer position, and bottom black line indicates the expected PCR product sizes. (**B**) Agarose gel image showing the *5*′ and *3*′ integration (5′ int—5′ integration and 3’ int—3′ integration) specific diagnostic PCRs (left panel), confirming the replacement of *Pbron6 orf* with SM (middle panel). The drug-resistant population of *PbΔron6* was subjected to cloning by limiting dilution from two independent transfections. Agarose gel image showing amplification of product only from the genomic DNA of wild type but not from *PbΔron6* clones cl1 and cl2 (right panel). Primer sequences and expected PCR product sizes are shown in Table S1. (**C**) Asexual growth propagation of *PbΔron6* cl1 and cl2 in BALB/c mice. 1 × 10^3^ infected RBCs of WT, *PbΔron6* cl1, and cl2 were injected intravenously in all three groups of BALB/c mice (*n* = 5/group). Parasitemia was monitored by reading the Giemsa-stained blood smears. Error bars represent the mean with standard deviation (**P < 0.005, one-way ANOVA with Tukey’s multiple comparison test). (**D**) Reinvasion assay was performed *in vivo* by intravenous injection of 1 × 10^9^ purified schizonts of WT and *PbΔron6* cl1 into two groups of BALB/c mice (*n* = 5). Parasitemia was monitored every 6 h post-infection by reading the Giemsa-stained blood smears. Error bars represent the mean with standard deviation (**P = 0.0045, Mann-Whitney test). (**E**) Kaplan-Meier plots, representing the duration of C57BL/6 mice survival, following infection with WT and *PbΔron6* cl1 (*n* = 10, ***P < 0.0001, Mantel-Cox test). (**F**) Strategy for complementation of *PbΔron6* locus. The top panel shows h*dhfr* and y*fcu* cassettes at *PbΔron6* locus. The middle panel shows an engineered complementation construct, carrying *Pbron6 orf* flanked by its native *5*′ and *3′ utrs*. The solid black lines indicate regions selected for homologous recombination. The lower panel shows complementation of the knockout locus with *Pbron6 orf*. Complemented parasites were enriched by negative selection with 5-fluorocytosine (5-FC). (**G**) Agarose gel image showing the presence of *Pbron6 orf*, *5*′ and *3*′ integration specific diagnostic PCRs confirming the replacement of SM cassette with *Pbron6 orf* (left panel). (**H**) Restoration of asexual propagation rate in *Pbron6* complemented (*Pbron6*(c)) clones—cl1 and cl2. *Pbron6* complemented (*Pbron6*(c)) parasites (cl1 and cl2) showing similar asexual propagation compared to WT (*P* = 0.9910, one-way ANOVA with Dunnett’s multiple comparison test). (**I**) Percentage of mice showing pre-patency. *Pbron6* complemented line shows similar pre-patency compared to WT in C57BL/6 mice (*n* = 5) (*P* > 0.99, Mantel-Cox test).

To investigate the functional role of *Pb*RON6 across all the life cycle stages of *Plasmodium*, a knockout mutant (*PbΔron6*) was generated by double homologous recombination. The *Pbron6 orf* was replaced with h*dhfr*::y*fcu* dual selectable marker (SM) cassette (Fig. 6A). The correct integration was confirmed by diagnostic PCRs that showed expected products of 1.6 and 1.2 kb that confirmed the 5′ and 3′ integrations, respectively (Fig. 6B left panel). The *Pbron6* null mutants were cloned by limiting dilution, and clones from two independent transfections were isolated for further phenotypic characterization. Genotyping of the clonal population by PCRs confirmed the absence of *Pbron6 orf* in clonal lines. (Fig. 6B, right panel).

Asexual propagation of *PbΔron6* line in mice was determined by delivering 1 × 10^3^ mutant parasites intravenously. Mice receiving a similar number of wild-type (WT) parasites served as a control. We observed a gradual increase in parasitemia of the WT line from approximately 4.5% to 17% during days 5–8. However, the parasitemia of mice harboring *PbΔron6* mutants did not exceed more than 1% during the same duration. The slow growth phenotype of the mutant was manifested identically in two null mutant clones derived from independent transfections (Fig. 6C). The data provided compelling evidence for the requirement of *Pbron6* in normal propagation of asexual stages. Considering that *Pbron6* showed high expression in the mixed blood stages, lack of expression may impact either the growth of the parasite or the reinvasion of freshly egressed merozoites, which accounted for its slow growth. To test this hypothesis, we injected intravenously 1 × 10^9^ synchronized schizonts of *PbΔron6* or the WT line into Swiss mice. Parasitemia was monitored every 6 h for a duration of 48 h. This allowed capturing reinvasion of freshly egressed merozoites into RBC, thus enabling the analysis of any defect in this process. We noted a progressive increase in parasitemia from 0.4% to 1.5% in WT. During this time, the parasitemia of the mutant line increased only in an incremental manner from 0.2% to 0.5% (Fig. 6D). Our observation reiterated that the reduced asexual propagation noted in earlier experiments was indeed associated with reduced invasion efficiency of erythrocytic merozoites. A likely explanation for the observed phenotype is that *Pb*RON6 may be required for the formation of the MJ, and its altered composition in the absence of *Pb*RON6 may dramatically impact but not totally abrogate invasion.

### Mice infected with *PbΔron6* parasites showed an enhanced survival rate

As mutant parasites lagged in asexual propagation, we next assessed the survival rate of mice harboring these mutants. To this end, we injected intravenously 1 × 10^3^ *PbΔron6* or WT parasites in female C57BL/6 mice (*n* = 10 per group). The survival duration of mice infected with WT parasites ranged from 8 to 16 days, while that of mice harboring *Pbron6* null mutants was significantly prolonged and survived for 22–32 days post-infection (Fig. 6E). Taken together, *Pb*RON6 depletion affected both the reinvasion capacity and overall virulence of the mutant parasite.

### *PbΔron6* complementation restored asexual propagation

To demonstrate that the observed phenotype in *PbΔron6* mutant was indeed due to the lack of *Pbron6* expression, we complemented the KO locus with *Pbron6 orf*. The complementation construct was generated by replacing the h*dhfr*::y*fcu* marker cassette in the *PbΔron6* construct with *Pbron6 orf*. The complementation construct was transfected into the *PbΔron6* mutant following standard transfection procedures, and the integrants were selected by providing 5-fluorocytosine (5-FC) in the drinking water of mice (Fig. 6F). The correct integrations were confirmed by diagnostic PCRs that showed expected products of 1.6 kb of both the 5′ and 3′ integrations (Fig. 6G). The *Pbron6*-complemented parasites (*Pbron6*(c)) were cloned by limiting dilution, and two clonal lines obtained from independent transfections were used for further phenotypic characterization. Genotyping of clonal population by PCRs using *Pbron6* primers confirmed the presence of *Pbron6 orf* in clonal lines (Fig. 6G).

With the complemented parasites, we analyzed the asexual propagation in mice. We injected 1 × 10^3^ asexual parasites of either WT or *Pbron6* complemented lines into Swiss mice by *i.v*. and monitored parasitemia from day 5 to 8. We noted that both complemented clones propagated at rates comparable to the WT (Fig. 6H) restoring the defect seen in the mutants. We also monitored the survival rate of mice in one of the complemented lines (clone 1). We noted that all five mice succumbed to mortality between 8 and 12 days, as noted for the WT line (Fig. 6I). Thus, complementation of *Pbron6 orf* in the KO line resulted in restoring the infectivity and virulence of the parasite.

### The *PbΔron6* parasites exhibited normal development in the mosquito vector

**Fig 7:**
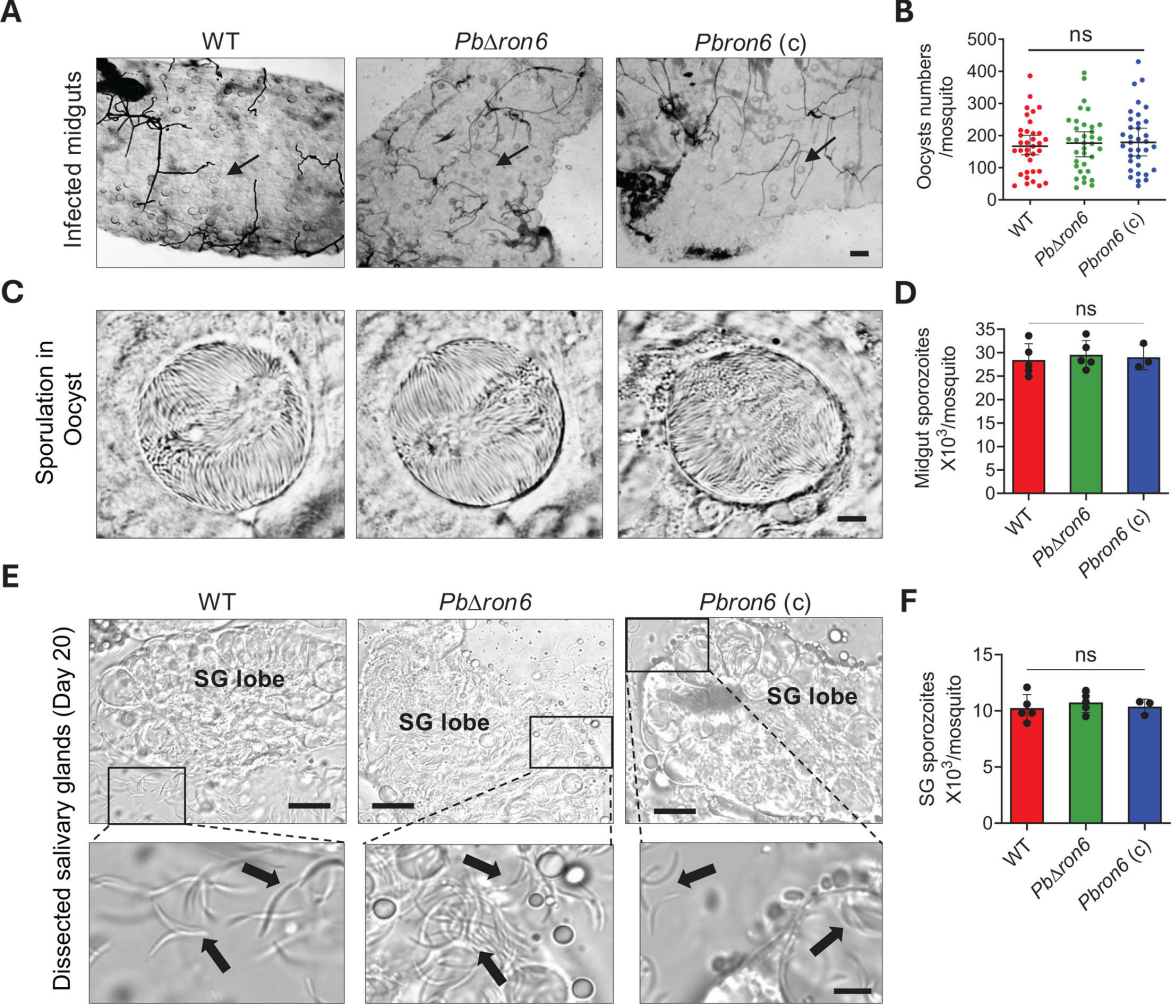
Phenotypic characterization of *PbΔron6* and *Pbron6* complemented (C) lines in mosquito. (**A**) Representative images of mosquito midguts, showing oocysts in WT, *PbΔron6*, and *Pbron6*(c) line. Scale bar: 200 µm. (**B**) The frequency of oocysts per mosquito in WT, *PbΔron6*, and *Pbron6*(c) lines. The horizontal lines indicate the median with 95% confidence interval (CI) (*n* = 35). Statistical differences were determined by one-way ANOVA with Holm-Šídák’s multiple comparisons test. ns: not significant. (**C**) Representative images of oocysts from WT, *PbΔron6*, and *Pbron6*(c) lines. Scale bar: 20 µm. (**D**) Bar graph showing the average number of oocyst sporozoites from WT, *PbΔron6*, and *Pbron6*(c) lines. Each bar represents the mean with standard deviation. *n* = 5 for WT, and *PbΔron6*, and *n* = 3 for *Pbron6*(c) line with 15–20 mosquitoes per experiment. Statistical differences were determined by one-way ANOVA with Tukey’s multiple comparisons test. ns: not significant. (**E**) Dissected salivary glands harboring sporozoites of WT, *PbΔron6*, and *Pbron6*(c) lines (top panel). Inset showing enlarged image of sporozoites (bottom panel). Scale bar: 200 µm. (**F**) Bar graph showing the average number of salivary gland sporozoites from WT, *PbΔron6*, and *Pbron6*(c) line. Each bar represents the mean with standard deviation. *n* = 5 for WT and *PbΔron6*, and *n* = 3 for *Pbron6*(c) line with 50–60 mosquitoes per experiment. Statistical differences were determined by one-way ANOVA with Tukey’s multiple comparisons test. ns: not significant.

To investigate the effect of the *Pbron6* depletion in the sexual stage of the parasite life cycle, female *A. stephensi* mosquitoes were infected with WT, *PbΔron6* mutant, or *Pbron6*(c) line. On day 14, post-blood meal, the mosquito midguts were observed for the presence of oocysts (Fig. 7A). We noted the oocyst numbers (Fig. 7B), sporulation pattern (Fig. 7C), and midgut sporozoite numbers (Fig. 7D) in the *PbΔron6* mutant line were comparable to both WT and *Pbron6*(c) lines. Since oocyst formation in the mutant is normal, this reiterated no discernible effect on ookinete formation. This observation concurred with the general notion that ookinetes that do not have rhoptries (15) remain unaffected when the composition of the rhoptry compartment is altered.

We next analyzed the ability of the mutant sporozoites to colonize the SGs. We noted the presence of *PbΔron6* sporozoites in mosquito SGs, whose morphology (Fig. 7E) and numbers (Fig. 7F) were indistinguishable from WT and *Pbron6*(c) lines. Taken together, the mosquito stages of *PbΔron6* manifested no alteration in phenotype, revealing a dispensable role of *Pb*RON6.

### *PbΔron6* mutant parasites showed delayed pre-patency

We next tested the effect of *PbΔron6* sporozoite infectivity on pre-patency, which is defined as the time required for detection of blood stage infection following delivery of infectious sporozoites. In two independent experiments, female C57BL/6 mice were infected intravenously with 5 × 10^3^ sporozoites of either *PbΔron6* or WT parasites. Parasitemia was monitored in all the animals from day 3 post-infection by making Giemsa-stained tail blood smears. Animals exposed to WT sporozoites exhibited pre-patency of day 3.5, whereas mice infected with *PbΔron6* mutant sporozoites, became pre-patent by day 8 (Table 1). When the experiment was repeated with 1 × 10^4^ sporozoites, we noted a pre-patency of 7.5 days in *PbΔron6* mutant, whereas WT-infected mice showed 3.5 days (Table 1).

**TABLE 1:** Analysis of prepatent period for *PbΔron6* sporozoites

Experiment #	Parasite strain	No. of animals	Sporozoite dose	Positive for blood stage infection	Pre-patency period (in days)
Expt 1	WT	6	5 × 10^3^	6	3.5
	*PbΔron6*	6	5 × 10^3^	6	8
Expt 2	WT	7	5 × 10^3^	7	3.5
	*PbΔron6*	7	5 × 10^3^	7	8
Expt 3	WT	3	1 × 10^4^	3	3.5
	*PbΔron6*	3	1 × 10^4^	3	7.5

### The *PbΔron6* mutant sporozoites exhibited normal gliding motility

**Fig 8:**
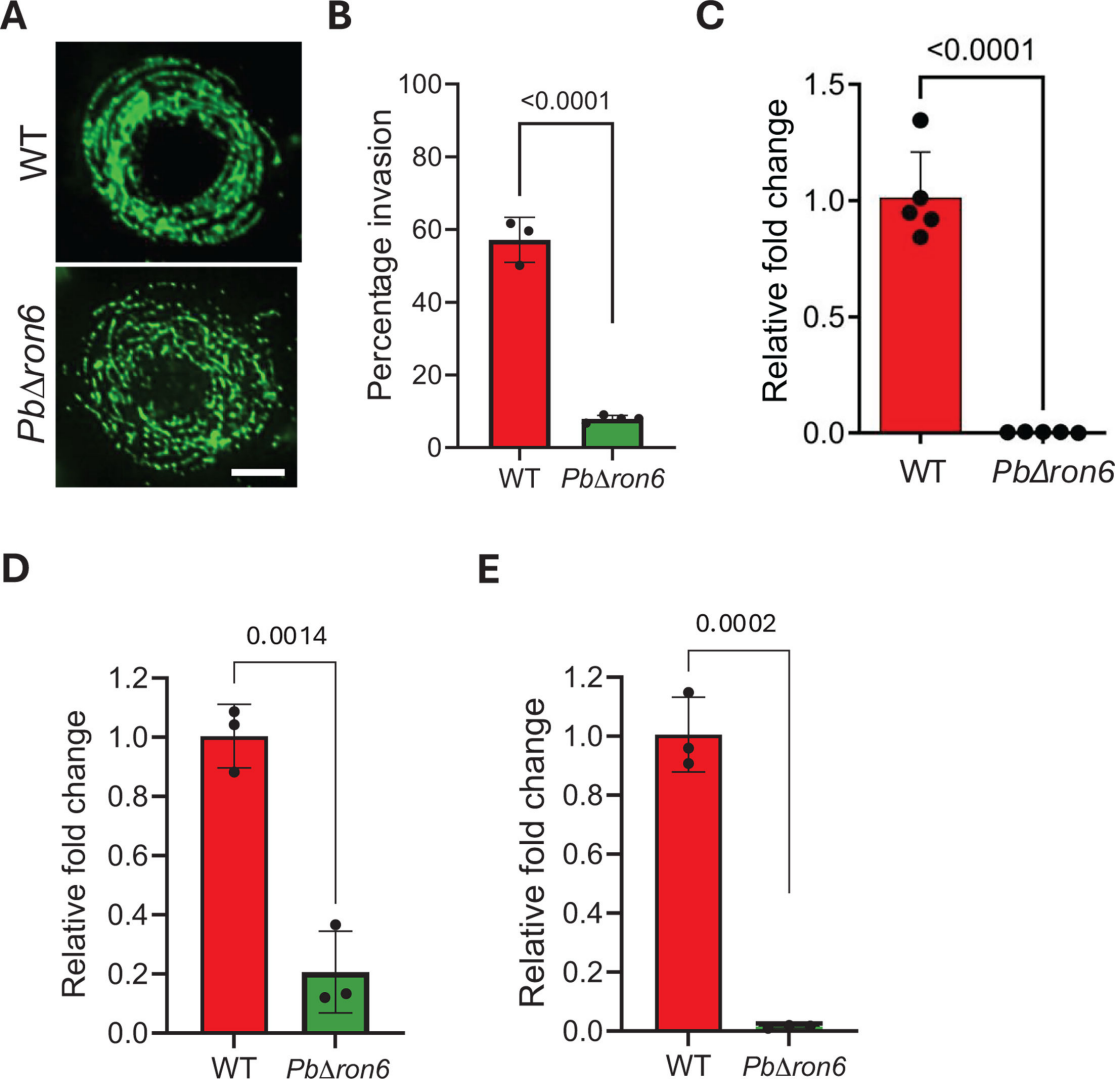
Assessing the *in vivo* infectivity of *PbΔron6* sporozoites. (**A**) Trails of CSP shed by *PbΔron6* sporozoites reveal normal gliding motility, comparable to WT. Scale bars: 10 µm. (**B**) Bar graph showing the invasion efficacy of *PbΔron6* in HepG2 cells quantified by the sporozoite inside-out assay. Each bar represents the mean with standard deviation. *n* = 3 for WT and *n* = 4 for *PbΔron6,* and 20 representative fields were counted for each experiment. Statistical difference was determined by unpaired *t*-test. ****P < 0.0001. (**C and D**) Bar graphs showing *P. berghei* 18S rRNA liver burden in mice at 6 h (**C**), 12 h (**D**), and *P. berghei MSP1* at 48 h (**E**). Mice were infected intravenously with 5 × 10^3^ sporozoites of the WT and *PbΔron6* cl1 line. Mice from each group were sacrificed, respectively, at 12 and 48 h. Total RNA was extracted from the livers of infected mice, and cDNA was generated. Real-time PCR was used to quantify the *P. berghei 18S* rRNA and *P. berghei MSP1* transcripts following 2^−ΔΔCt^ method. Mouse *gapdh* was used for the normalization. Each bar represents the mean with standard deviation and *n* = 3, with each experiment performed in triplicate. Statistical difference was determined by an unpaired *t*-test.

*PbΔron6* sporozoites exhibited delayed pre-patency compared to WT sporozoites, which is likely attributable either to a defect in gliding motility or hepatocyte invasion. *Plasmodium* sporozoites rely on substrate-dependent, actin-based gliding motility to penetrate host cells and traverse cellular barriers (47). The gliding motility of SG sporozoites was analyzed by observing the patterns of CSP trails. *PbΔron6* sporozoites displayed CSP trails similar to WT sporozoites, indicating normal gliding motility (Fig. 8A).

### *Pb*RON6 is required for the host cell invasion

Since erythrocytic merozoites were compromised to invade RBCs under conditions of *Pbron*6 depletion, we next investigated if other invasive stages, like the SG sporozoites, had any discernible deficiency in hepatocyte invasion. To test this, we added 2 × 10^4^ sporozoites of either WT or *PbΔron6* mutants to HepG2 monolayer and maintained for 1 h at 37°C, prior to fixation. A dual sporozoite staining assay using 3D11 (anti-CSP) monoclonal antibody under permeabilized and non-permeabilized conditions, called the “inside-out assay,” was performed (48). The assay allowed quantification of invaded and extracellular sporozoites that revealed 60% invasion of the WT sporozoites, while the *PbΔron6* sporozoites exhibited invasion efficiency of nearly 20% (Fig. 8B). We conclude that the mutant sporozoites failed to invade HepG2 cells efficiently in the absence of *Pb*RON6.

### *Pbron6* depletion altered sporozoite infectivity and EEF development

To investigate the *in vivo* hepatocyte invasion efficiency, the parasite liver burden was estimated in mice infected with *PbΔron6* or WT parasites. C57BL/6 mice were injected with 5 × 10^3^ sporozoites intravenously, and livers were isolated at 6, 12, and 48 h post-infection (hpi). Total RNA was isolated from the livers, and approximately 2 µg was reverse transcribed. cDNA samples were used to quantify the expression of *Pb18S rRNA* at 6 and 12 h, and *msp1* at 48 h by qRT-PCR. Mouse *gapdh* was used as an internal control. The mutants manifested nearly 99-fold and 5-fold reduction in sporozoite commitment to hepatocytes as inferred from the *Pb18S rRNA* quantification at 6 and 12 h, respectively (Fig. 8C and D). We also noted a 65-fold reduction in liver burden quantified from *msp1* expression at 48 hpi (Fig. 8E). Taken together, *Pbron6* depletion resulted in reduced efficiency of sporozoite invasion and also developmental arrest in mutant EEFs. The developmental arrest in the liver may likely be attributed to the lack of association of *Pb*RON6 with PVM in the developing EEFs in mutants.

**Fig 9:**
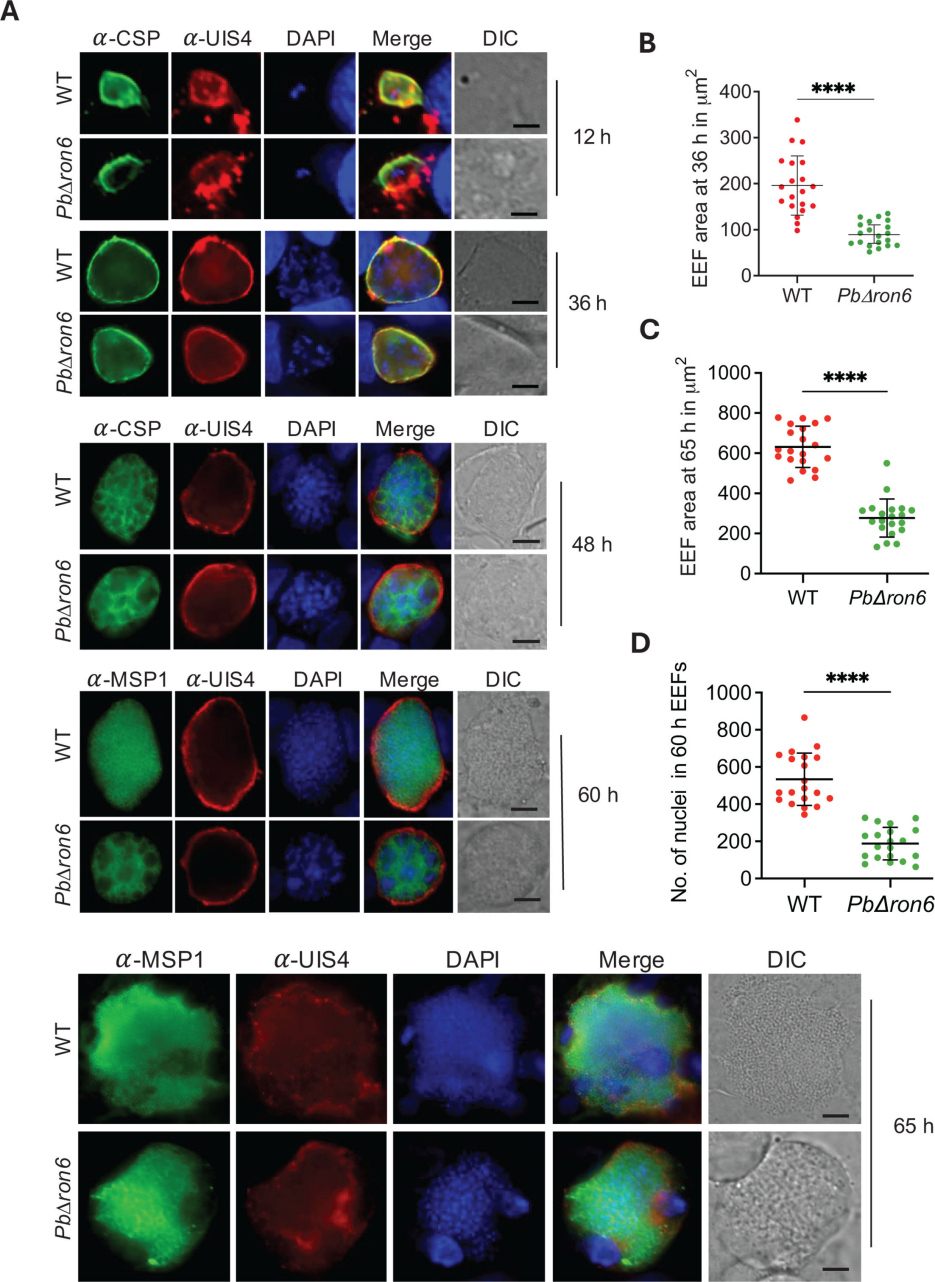
EEF development of *PbΔron6* mutants in HepG2 cells. (**A**) Growth of EEFs was monitored at 12, 36, 48, 60, and 65 h post-invasion, by performing an immunofluorescence assay. The 12, 36, and 48 h cultures were stained with rabbit anti-UIS4 (PVM marker) and mouse anti-CSP (3D11). Immunoreactivity was revealed, respectively, with Alexa Fluor 594-conjugated anti-rabbit and Alexa Fluor 488-conjugated anti-mouse secondary antibodies. The 60 and 65 h cultures were stained with rabbit anti-UIS4 antibody and mouse anti-MSP1 antibodies. Immunoreactivity was revealed, respectively, with Alexa Fluor 594-conjugated anti-rabbit and Alexa Fluor 488-conjugated anti-mouse secondary antibodies. DAPI was used to visualize host and parasite nuclei. Scale bar: 10 µm. (**B and C**) Dot plots showing the quantification of EEF area at 36 and 65 h post-infection using NIS-Elements AR software. Error bars represent the mean with the standard deviation. Statistical difference was determined by Mann-Whitney test. ****P < 0.0001. (**D**) Dot plot showing the quantification of hepatic merozoites in 65 h EEFs. Error bars represent the mean with the standard deviation. Statistical difference was determined by Mann-Whitney test. ****P < 0.0001.

Owing to the delay in pre-patency and reduced *msp1* levels in mice exposed to *PbΔron6* sporozoites, we next analyzed if there was any developmental defect associated with EEF maturation. To test this, the HepG2 monolayers were infected with 1.5 × 10^4^ sporozoites of either WT or *PbΔron6* sporozoites, and the cultures were fixed at different time points of EEF maturation. The liver stage cultures were stained with a PVM marker, UIS4, and EEF marker CSP (Fig. 9A) at 12, 36, and 48 h. The 60 and 65 h EEFs were stained with MSP1 and UIS4 (Fig. 9A). While no detectable size difference was observed at 12 h in *PbΔron6* mutant EEFs as compared to WT, in all the other developmental stages, we found that EEFs derived from mutant sporozoites were smaller than WT (Fig. 9B and C). We also observed a notable reduction in hepatic schizogony at the 65-h time point (Fig. 9D). Interestingly, the cytomeres within the *PbΔron6* mutant EEFs were larger as compared to WT at 48 and 60 h, indicating a developmental defect (Fig. 9A). A small fraction of mutant EEFs appeared similar in size to WT EEFs; however, they were still in the cytomere stage, indicating a lag in the development (Fig. S3B and C).

### *PbΔron6* parasites induced hyper-reactive malarial splenomegaly

**Fig 10:**
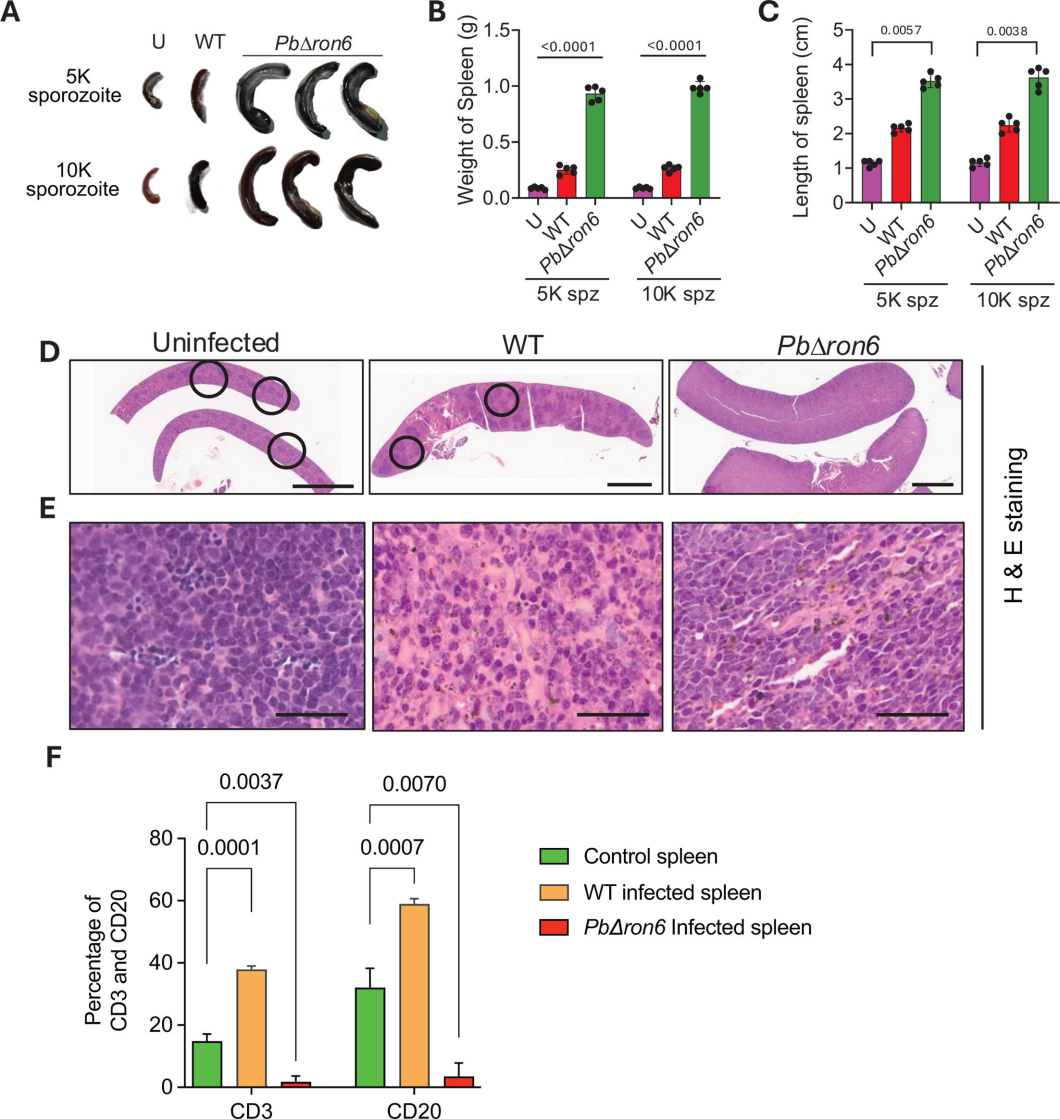
Histopathology of spleens taken from *PbΔron6*-infected mice reveals hyper-reactive malarial splenomegaly. (**A**) Representative images of spleens isolated from uninfected (U), *P. berghei* wild-type (WT), and *PbΔron6*-infected mice. The upper panel and lower panel show images of spleens taken from C57BL/6 mice exposed to an intravenous dose of 5 × 10^3^ and 1 × 10^4^ sporozoites, respectively. (**B**) Bar graph showing the weight of spleens measured in grams from uninfected (U), *P. berghei* WT, and *PbΔron6*-infected mice. Statistical differences were determined by one-way ANOVA with Tukey’s multiple comparisons test (*n* = 5). (**C**) Bar graph showing the lengths of spleens measured in cm from uninfected (U), *P. berghei* WT, and *PbΔron6*-infected mice. Statistical differences were determined by the Kruskal-Wallis test with Dunn’s multiple comparisons test (*n* = 5). (**D**) H&E staining of spleen sections from uninfected, *P. berghei* WT and *PbΔron6*-infected mice. The black circles indicate intact lymphoid follicles in the spleens. No lymphoid follicles were noted in the spleens of *PbΔron6*-infected mice. Scale bar: 2 mm. (**E**) Sections of spleen showing hemosiderophages in uninfected, *P. berghei* WT, and *PbΔron6*-infected mice. Scale bar: 30 µm. (**F**) Bar graph showing the B and T cell quantification by flow cytometry. The splenocytes obtained from uninfected, *P. berghei* WT, and *PbΔron6*-infected mice were stained with anti-CD3 and anti-CD20 and quantified by flow cytometry.

Splenomegaly is a frequent clinical manifestation of malaria infection (49). We observed an increase in size (Fig. 10A), weight (Fig. 10B), and length (Fig. 10C) of spleens taken from the mice infected with *PbΔron6* compared to WT. The average weight of the uninfected spleens was nearly 89 mg, while spleens taken from WT-infected mice weighed nearly 260 mg. Interestingly, the spleen from mice infected with the *PbΔron6* mutant weighed approximately 960 mg. The increased weight of spleens in mice infected with *PbΔron6* mutants was reminiscent of hyper-reactive malarial splenomegaly (HMS) that appears sporadically in humans subjected to chronic malaria infections. We noted that the ability to induce HMS in C57BL/6 mice was independent of the sporozoite inoculum used to induce infection (Fig. 10A through C).

To investigate the histopathological changes in the spleens of mice infected with *PbΔron6* mutants, H&E staining was performed. A clear demarcation between the white pulp and red pulp, with prominent marginal zones and resting follicles, was observed in the spleens of uninfected mice. However, in the spleens of WT-infected mice, an enhanced cellularity in red pulp and an increased size of white pulp were observed. Interestingly, in the spleens of mice infected with *PbΔron6* mutants, there was an obliteration of the white pulp region with predominance of red pulp and indistinct demarcation of marginal zones (Fig. 10D). An increased number of hemosiderophages in spleens obtained from mutant-infected mice indicated the over-destruction of iRBC (Fig. 10E). Immunophenotypic analysis of spleens was performed by analyzing the levels of CD20, a B-cell marker, and CD3, a T-cell marker, by flow cytometry. We noted that in *PbΔron6*-infected mice, both CD20 and CD3 expressing cells were low as compared to mice infected with WT or no infection (Fig. 10F). This concurred with the loss of lymphoid follicular patterns in *PbΔron6*-infected mice. Taken together, hyper-reactive malarial splenomegaly induced by *PbΔron6* mutants dramatically reduced the population of B and T lymphocytes in the spleen, which hints at an altered immunological niche.

## DISCUSSION

In the current study, an in-depth functional investigation of *Pb*RON6, a putative orthologue of *P. falciparum* RON6, was performed across all life cycle stages using a genetic approach. These studies revealed novel and previously unappreciated roles of *Pb*RON6 in cellular redistribution, host cell invasion, and maintenance of virulence. Structurally, *Pb*RON6 lacks the C-terminal Cys-rich domain, previously implicated in host cell invasion and correct protein trafficking in *P. falciparum* (50). Further, in striking contrast to the indispensable nature of the *Pfron6* locus and its C-terminal Cys-rich domain (50), we demonstrate the feasibility of successfully targeting the *Pbron6* locus.

Bioinformatics and biochemical approaches revealed localization of *Pf*RON6 to rhoptries in schizonts and rings and to PVM in maturing intra-erythrocytic forms (50). However, not much is known about the localization of *Pf*RON6 in the sporozoite and EEFs. The only studies available for RON6 localization are from the rodent malaria model of *P. berghei*, using an immunoelectron microscopy approach, where it was shown to reside in rhoptries of sporozoite (51). However, the cellular distribution of *Pb*RON6, especially in the developing liver stages and merosomes, is yet to be characterized. We used online prediction tools to assess the cellular probability of RON6. DTU/Deep TMHMM predicted both *P. falciparum* and *P. berghei* RON6 as globular proteins. Both orthologs lack a transmembrane domain but possess a large extracellular domain. Phobious server predicted both *Pf*RON6 and *Pb*RON6 as non-cytosolic proteins. Consistent with these predictions, we noted an association of *Pb*RON6::3xHA with the sporozoite and PVM membrane, colocalizing with CSP and UIS4, respectively. Interestingly, the extracellular nature of *Pb*RON6 C-terminal domain could also be confirmed by HA immunoreactivity on sporozoites under non-permeabilized conditions. Previous studies reported the immunoreactivity of malarial antisera from Vietnam and Papua New Guinea to the N, C, and Cys-rich repeat regions of recombinantly expressed *Pf*RON6 (50), likely implying that all three domains are targets for antibodies in the merozoite stage. The positive immunoreactivity towards *Pf*RON6 may possibly be explained, owing to the presence of a signal peptide that allows its trafficking to the secretory compartment, as demonstrated earlier (50). Though DeepTMHMM predicted a signal peptide in *Pb*RON6, N-terminal antisera from *Pb*RON6 (110–281 AAs) failed to show immunoreactivity on sporozoite membrane under non-permeabilized condition. Rather, we noted an intracellular localization, most prominently in proximity to the nucleus, likely hinting to an ER association that needs further confirmation. It is speculative if *Pb*RON6 targets to ER and only processed form translocates to the sporozoite membrane, with a possible C-terminus positioned extracellularly.

The punctate staining pattern of *Pb*RON6 in daughter cells of schizonts and hepatic merozoites, characteristic of the rhoptries, is by and large, expected. This may explain the lack of optimal invasion of *Pbron6* mutants into RBC, given that rhoptry neck proteins have been implicated in the formation of MJ (52), though a definitive role of *Pb*RON6 in the complex has not been demonstrated in this study. Our study also provides additional evidence for the role of *Pb*RON6 in the invasion of hepatocytes, both under *in vitro* and *in vivo* conditions. Following sporozoite inoculation into a mammalian host, and prior to its hepatocyte entry, several transcripts implicated in invasion and subsequent intrahepatic development are upregulated, as demonstrated in a model of co-culture of *P. falciparum* sporozoites with primary human hepatocytes (53). This concurs with the “just in time” model of gene expression, preparing invasive stages for host cell entry, characterized by enhanced synthesis of apical secretory and parasite surface proteins (54). The maximal gene expression of *Pbron6* in schizonts and SG sporozoites may imply two bursts of transcriptional activities, coinciding with the need to invade RBC and hepatocytes respectively.

Interestingly, we noted aggregates of *Pb*RON6::3xHA at multiple foci over the membrane when sporozoites contacted with host cells maintained at 37°C. This hinted at its role in hepatocyte commitment, though no secreted form of *Pb*RON6::3xHA was detected in sporozoite incubated supernatants. In comparison to the rhoptry discharge that occurs only once during the RBC invasion of merozoites, sporozoites may have to release the rhoptry contents, possibly during SG invasion, hepatocyte invasion, and also export to the EEF membrane. Therefore, PbRON6 secretion may be programmed for release in multiple bouts to meet the aforementioned invasion and EEF development-related functions.

A conditional depletion of RON 2, 4, and 5 complexes affects normal substrate attachment in sporozoites, resulting in reduced gliding (36). A similar function was also noted for *Pb*RON11 (35). Contrastingly, we noted no effect of *Pb*RON6 depletion on the colonization of sporozoites in the SGs, substrate attachment, and gliding. However, mutant sporozoites showed a seven fold reduction in HepG2 cell invasion as compared to WT, which was also reiterated *in vivo*, as noted from reduced levels of *Pb18S* rRNA at 6 and 12 hpi, following exposure of mice to mutants. Taken together, these observations point to the role of *Pb*RON6 in hepatocyte invasion.

A reduction in hepatic schizogony was another important observation noted in *PbΔron6* mutants. It is speculative whether the reduction in hepatic schizogony is due to a lack of recruitment of *Pb*RON6 on PVM. In fact, other proteins like RAP2/3 that localize to rhoptries of midgut and SG sporozoites are recruited to PVM (55). Another rhoptry protein, ICP, which has a role in the inhibition of host proteases and egress of hepatic merozoites (56), also continues to be expressed in *P. yoelii* sporozoite cytoplasm and in PVM of *P. yoelii* and *P. falciparum*. While the precise role of *Pb*RON6 in PVM maintenance is yet to be deciphered, decreased schizogony, reduction in the *msp1* expression at 48 h, and delay in pre-patency reiterated a growth defect during late EEF development.

The IP and mass spectrometric analysis with C-terminal HA-tagged *Pb*RON6 parasites revealed *Pb*RON6 interaction with a diverse range of putative proteins involved in erythrocyte invasion and host cell remodeling. This may explain the delay in asexual propagation and RBC reinvasion associated with the mutant. Of particular interest were proteins like MSP1, MSP9, RON3, RAP1, and Apical merozoite protein, an ortholog of *Pf*34, that were detected only in pulldown with *Pb*RON6::3xHA parasite lysates. MSP1 forms a complex with other merozoite surface proteins such as MSP3, MSP6, MSP7, and MSP9 (57) to facilitate anchoring to the erythrocyte membrane via band3 protein (58). Paradoxically, however, merozoites lacking MSP1 expression can still invade RBCs (59), and a likely mechanism may be by utilizing peripheral invasins, which may mediate tight attachment to the RBC surface. Though not demonstrated in merozoites, our study shows an association of the *Pb*RON6 C-terminal extracellular domain with the sporozoite membrane that may act as an invasin in the invasion complex. Another novel rhoptry bulb protein detected in LC/MS analysis with a high confidence score was RON3. A recent work claimed that knockdown of *Pf*RON3 C-terminal fragment expression in the early schizont stage induced a defect in erythrocyte invasion and resulted in developmental arrest at the ring stage (60, 61). We observed a similar phenotype where reinvasion was compromised in *Pb*RON6 mutants. Further, two independent studies elucidating colocalization of *Pf*RON6 with *Pf*34 in the rhoptry neck (50) and interaction of C-terminus of *Pf*RON6 with *Pf*34 (62), aligns with our findings from LC/MS-based interactome studies.

A massive increase in spleen size during malaria infection, referred to as hyper-reactive malarial splenomegaly (HMS) (63), occurs due to chronic antigenic stimulation in subjects having long-term exposure to malaria parasites (64). Although a triad of symptoms characterized by enlarged spleen, raised IgM levels, and the presence of malaria antigens are hallmarks of HMS, the condition is highly variable (65). The *PbΔron6* mutant induces HGM and offers a model to investigate the pathological insights of the condition. Our preliminary studies revealed reduced numbers of B and T lymphocytes in spleens obtained from mice infected with *PbΔron6* mutants. It is speculative if the excess hemozoin associated with hemosiderophages, as seen in this study, alters the expression of MHC class II, costimulatory, and adhesion molecules together with inhibition of dendritic cell maturation (66–68). This may explain why the parasites fail to get cleared in spite of their slow progression in mice.

Two independent studies employing conditional silencing of *Pf*RON6 have shown their role in asexual propagation (62, 69), mirroring our current observations. In the first study, a knock-sideways approach was successfully used to inactivate *Pf*RON6 within the ER through fusion of the KDEL-ER retrieval sequence (69). In a second study, an AVEXIS assay was employed to characterize the interactions of human and *P. falciparum* sporozoite proteins, involved in host cell invasion (62). This study reiterated the interaction of the C-terminus of *Pf*RON6 with *Pf*34, which concurs with earlier demonstrations of their colocalization in the rhoptry neck (50). Interestingly, no interaction of *Pf*34 was noted with the N-terminal domain of *Pf*RON6 (62). This coincides with our observation where the antisera raised against recombinant PbRON6^110–281^ showed intracellular localization around the nucleus, likely in the ER compartment (Fig. S4A through E).

An invasion and developmental defect associated with *Pb*RON6 depletion was indeed a locus-specific effect, as complementation restored asexual propagation, pre-patency, and virulence. With the advancement in our understanding of the secretory pathways and invasion mechanisms of apicomplexan parasites, it is becoming increasingly clear that some components of the invasion machinery are conserved between erythrocytic and sporozoite stages. Given that rhoptry protein secretion critically regulates cell traversal, host cell invasion, PVM formation, and proliferation of parasites within the PV, targeting the secretory effectors may prevent malaria transmission at multiple life cycle stages. In line with this idea, we demonstrate the role of *Pb*RON6 in the parasite invasion of RBC, hepatocytes, and its association with sporozoite and PV membranes. Thus, targeting *Pb*RON6 may enhance the breadth of beating the parasite at multiple life cycle stages for efficient control of malaria.

## MATERIALS AND METHODS

### Experimental animals

Female Swiss albino, BALB/c, and C57BL/6 mice aged 6–8 weeks old were used. The animals were procured from the National Centre for Laboratory Animal Sciences, National Institute of Nutrition, Hyderabad, and Hylasco Bio-Technology (India) Pvt. Ltd., Hyderabad. The experimental animals were housed in a controlled environment at the animal facility of the University of Hyderabad. The animals were maintained at 22°C, with a relative humidity of 50–60%, and 12-h dark-light cycle. A standard diet as prescribed for rodents was provided *ad libitum*. All the animal protocols conducted in this study were approved by the Institutional Animal Ethics Committee of the University of Hyderabad.

### Parasite lines

A rodent species of *Plasmodium berghei ANKA (P. berghei),* well adapted to the laboratory conditions, was used in this study. The WT *P. berghei* line is highly amenable to genetic modification and was used as a parental line to perform gene manipulations.

### Bioinformatic analysis

A *Plasmodium* database, PlasmoDB (https://plasmodb.org/plasmo/app) was used to retrieve the gene and AA sequences. The retrieval of gene identities and orthologs for other *Plasmodium* species was also from the same database.

The gene and protein sequences of *P. berghei ron6* (*Pbron6*, PBANKA_0311700), *P. falciparum ron6* (*Pfron6*, PF3D7_0214900), and from other *Plasmodium* species were retrieved from PlasmoDB. *Pb*RON6 and *Pf*RON6 were analyzed using the Deep TMHMM tool (https://dtu.biolib.com/DeepTMHMM) to predict the transmembrane topology of RON6. Similarly, Phobius web server was used for predicting the cellular distribution of the protein (https://phobius.sbc.su.se/). Signal P-4.1 was used to identify signal peptide sequences (https://services.healthtech.dtu.dk/services/SignalP-4.1/). The sequence alignment of RON6 orthologs from *Plasmodium* species was performed using Clustal Omega (https://www.ebi.ac.uk/jdispatcher/msa/clustalo) to identify conserved motifs.

### The expression analysis of *Pbron6*

Expression of *Pbron6* across the life cycle stages was analyzed by qRT-PCR following absolute quantification. Total RNA was isolated from rings, trophozoites, schizonts, midgut sporozoites, SG sporozoites, and *in vitro* liver stages at 12, 24, 36, 48, and 60 h post-infection (hpi), and merosomes of *P. berghei* using Ambion PureLink RNA Mini Kit (Thermo Scientific, Cat #12183020) following the manufacturer’s instructions. *Pbron6* gene-specific standards were generated by amplifying a 136 bp fragment that was ligated into a pTZ57R/T vector (Thermo Scientific, Cat #K1214). Similarly, a 154 bp product of *Pbef1α* ligated into the pTZ57R/T vector was used as an internal control. Primers used for qRT-PCR are shown in Table S1. Gene-specific standards were generated in log scale dilution of plasmid copy numbers ranging from 10^8^ to 10^2^. The RNA was subjected to DNase I treatment (NEB Cat #M0303S), and cDNA was synthesized from 2 µg of total RNA isolated from different stages of *P. berghei* using the PrimeScript 1st strand cDNA Synthesis Kit (Takara, Cat #61100A), following the manufacturer’s instructions. SYBR Green master mix (TAKARA TB Green Premix Ex TaqTM II Tli RNase H Plus, Cat #RR82R) was used for qRT-PCR. Real-time PCR was performed using Eppendorf RealPlex 2 (Cat #2894). *Pbron6* absolute transcript numbers were quantified at the selected life cycle stages by qRT-PCR using gene-specific primers run along with the gene-specific standards and normalized with the absolute transcript numbers of *Pbef1α*.

### Generation and genotyping of *Pb*RON6 HA transgenic and mutant lines

To localize *Pbron6* throughout the life cycle stages, a C-terminal hemagglutinin (3xHA) epitope-tagged *Pbron6::3xHA* line was generated. To achieve this, 455 bp *3*′ part of *Pbron6 orf* eliminating the stop codon and 507 bp of *Pbron6 3′ utr* (un-translated region) were amplified from *P. berghei* genomic DNA using the primers P1/P2 and P3/P4, respectively (Fig. 2A; Fig. S2A; Table S1 for details of the primers). The amplified partial *orf* and *3′ utr* were introduced into the XhoI/BglII and NotI/AscI restriction sites of pBC-3xHA-hDHFR plasmid (Fig. S2B) (70), respectively, to obtain the *Pbron6::3xHA* construct. *Pbron6::3xHA* plasmid contains h*dhfr* SM under the control of the constitutive *Pbeef1a* promoter. *Pbron6::3xHA* plasmid was linearized with restriction enzymes XhoI and AscI and transfected into *P. berghei* parasite line following standard transfection procedure (71). DNA-integrated parasites were selected by providing pyrimethamine in the drinking water of mice, resulting in the enrichment of the *Pbron6::3xHA* tagged line (Fig. 2A). The recombined locus having 3xHA in frame with *Pbron6 orf* was confirmed by Sanger sequencing (Fig. S2C) following PCR amplification using diagnostic primers P24/P11 (see Table S1 for details of the primers). The drug-resistant parasites were cloned by limiting dilution (72), and correct integration of the *Pbron6::3xHA* construct was confirmed by diagnostic PCR analysis from the genomic DNA of the cloned parasite lines (Fig. 2A). Details of the primers used for genotyping and expected PCR product sizes are listed in Table S1. Two clones that were generated from independent transfections were used for further analysis.

The *Pbron6* deletion (*PbΔron6*) construct was generated using the plasmid pSKC-hDHFR::yFCU, which carries a h*dhfr*::y*fcu* positive-negative SM under a constitutive *Pbeef1α* promoter (39). The *Pbron6* locus was targeted by the double-crossover homologous recombination method. To achieve this, the 5’ and 3’ untranslated regions (*utr*s) of *Pbron6* were amplified from *P. berghei* genomic DNA using the primer pairs P5/P6 and P3/P7, respectively (see Table S1 for details of the primers) and introduced into XhoI/HindIII and NotI/AscI restriction sites, respectively, to obtain *PbΔron6* construct. The *PbΔron6* construct was linearized with XhoI and AscI restriction enzymes and transfected into *P. berghei* parasite line following the standard transfection procedure (71). DNA-integrated parasites were selected by providing pyrimethamine in the drinking water of mice, resulting in the enrichment of *PbΔron6* line. The drug-resistant parasites were cloned by limiting dilution (72), and correct integration of the *PbΔron6* construct was confirmed by diagnostic PCR analysis from genomic DNA of the cloned parasite lines (Fig. 6A). Details of the primers used for genotyping and expected PCR product sizes are listed in Table S1. Two clones that were generated from independent transfections were used for further analysis.

For complementation of the *PbΔron6* locus, *Pbron6 orf* was amplified from *P. berghei* genomic DNA using the primer pairs P8/P9. *Pbron6 orf* was used to replace the h*dhfr*::y*fcu* cassette in *PbΔron6* construct using the restriction sites HindIII/NotI. *Pbron6* complementation construct was linearized with XhoI and AscI restriction enzymes and transfected into *PbΔron6* parasite line following standard transfection procedure (71). The complemented parasites were subjected to negative selection by providing 5-FC in the drinking water of mice. The drug-resistant parasites were cloned by limiting dilution (72), and correct integration of the *Pbron6* complementation construct was confirmed by diagnostic PCR analysis from genomic DNA of the cloned parasite lines (Fig. 6F). Details of the primers used for genotyping and expected PCR product sizes are listed in Table S1. Two clones generated from independent transfections were used for further analysis.

### Analysis of the *PbRON6* KO phenotype in the mixed blood stages

Cloned *PbΔron6* lines were injected into mice to monitor their asexual propagation rate. Approximately 1 × 10^3^ *PbΔron6* or WT parasites (control) were intravenously injected into five Swiss mice. The parasitemia was monitored by Giemsa-stained smears from day 3 of post-infection until day 8. The data was recorded from 25 fields/mouse on a daily basis, and the average percentage of parasitemia was calculated.

### Transmission of *Pbron6* mutant parasites to *Anopheles stephensi*

For mosquito transmission experiments, nearly 150–200 female *Anopheles* mosquitoes were fed on the anesthetized mice with circulating gametocytes of *Pbron6* mutants or WT parasites. The infected mosquitoes were maintained at 21°C with relative humidity of 75–80% and were supplemented with 5% sucrose.

### Analysis of oocyst development inside the mosquito

To study the phenotype of the *Pbron6* mutants, infected mosquito mid-guts were manually dissected using a dissection microscope (Lawerence & Mayo, Cat #NSZ-606) on day 14 post-infection. The number of oocysts were quantified, and sporulation patterns inside the oocysts were observed. For quantification of the oocyst sporozoite load, the dissected mosquito midguts were collected in a 1.5 mL tube and manually disrupted using a plastic pestle to release sporozoites. The released sporozoites were counted using a hemocytometer.

### Isolation of SG sporozoites

To isolate the SG sporozoites, the infected mosquitoes were dissected between days 18 and 21, post-infection. Mosquito SGs were manually dissected and collected in RPMI-1640 in a 1.5 mL tube. The SGs were disrupted using a plastic pestle, and the sporozoites were counted using a hemocytometer.

### HepG2 cell cultures for assessing *in vitro* EEF development

To investigate the developmental progression of the *Pbron6* mutant liver stages *in vitro*, parasite growth was assessed in HepG2 cells. Collagen-coated coverslips were seeded with HepG2 cells at a density of 1 × 10^5^ in a four-well plate (Thermo Scientific, Cat #144444), 1 day prior to the addition of sporozoites. On days 18–21 post-infection of mosquitoes, the *Pbron6* mutant sporozoites were isolated and approximately 2 × 10^4^ sporozoites were added to HepG2 monolayers and incubated at 37°C in a CO_2_ incubator. Infected cultures were fixed at 12, 24, 48, 60, and 65 hpi with 4% paraformaldehyde (PFA) (Thermo Scientific, Cat #043368.9M). For producing axenic EEFs, sporozoites were added to cover slips placed in four-well plates containing DMEM (Gibco, Cat #11965-092) supplemented with 10% fetal bovine serum (Gibco, Cat #10270-106). Six hours post-addition of sporozoites, the cultures were fixed with 4% PFA and processed for indirect IFA.

### IFA

The schizonts, gametocytes, midgut sporozoites, SG sporozoites, and developing *in vitro* liver stages (6, 12, 26, 36, 48, and 62 h) of *Pbron6::3xHA* line were fixed with 4% PFA, permeabilized with cold acetone and methanol (1:3), and washed with PBS. All samples were blocked with 3% BSA solution. For schizonts, gametocytes, midgut sporozoites, SG sporozoites, and EEFs at 36 hpi, primary antibody incubations were done with mouse anti-HA monoclonal antibody (Abcam, Cat #AB18181) at 1:100 dilution, along with respective stage-specific markers. The marker used for schizonts was rabbit anti-PIC5 antibody (Dey S et al., manuscript under preparation) at 1:100 dilution. Rabbit anti-SIMP (39) was used as a marker for gametocytes, midgut, and SG sporozoites at 1:100 dilution. Rabbit anti-UIS4 antibody (kind gift from P. Sinnis, JHU) was used as a marker for EEFs used at 1:1,000 dilution. Primary antibodies were removed after 1 h incubation, washed three times with PBS followed by incubation with Alexa Fluor 488-conjugated goat anti-mouse secondary antibody (Thermo Scientific, Cat #A11017) and Alexa Fluor 594-conjugated chicken anti-rabbit secondary antibody (Thermo Scientific, Cat #A21442) at 1:1,000 dilution. Nuclei were stained with DAPI (Sigma, Cat #D9564) along with secondary antibodies. 6 h axenic culture, *in vitro* liver stages at 12, 26, 36, 48, and 62 hpi, and merosomes were stained with rabbit anti-HA monoclonal (Abcam, Cat #AB236632), 3D11, or MSP1 antibodies. The anti-HA monoclonal antibody, 3D11 (73), and anti-MSP1 were used at 1:100, 1:1,000, and 1:5,000 dilutions, respectively. Primary antibodies were removed after 1 h incubation, washed three times with PBS. Immunoreactivity was revealed with Alexa Fluor 594-conjugated chicken anti-rabbit secondary antibody, and Alexa Fluor 488-conjugated goat anti-mouse secondary antibody at 1:1,000 dilution. Nuclei were stained with DAPI. The slides were mounted with a coverslip using ProLong Gold Anti-Fade Reagent (Thermo Scientific, Cat #P10144) and sealed with nail polish. The slides were visualized under a Nikon Eclipse upright fluorescence microscope, and images were captured using the NIS-Elements AR software.

To confirm the localization of *Pb*RON6 on the surface of the sporozoite, an IFA was performed with and without permeabilization. As described previously, *Pbron6::3XHA* sporozoites were blocked with 3% BSA solution. Two commercially procured anti-HA antibodies, namely anti-HA rabbit monoclonal (Abcam, Cat #AB236632) and anti-HA rabbit polyclonal (Abcam, Cat #AB9110), were used for IFA at 1:100 dilution. The 3D11 monoclonal antibody (73) used at 1:1,000 dilution served as a marker for staining the sporozoite membrane. The immunoreactivity of 3D11 and HA was revealed with Alexa Fluor 488-conjugated goat anti-mouse and Alexa Fluor 594-conjugated chicken anti-rabbit secondary antibodies at 1:1,000 dilution, respectively. The immunoreactivity was visualized under a Nikon Eclipse upright fluorescence microscope, and images were acquired and analyzed using NIS-Elements AR software.

The development of *PbΔron6* EEFs was studied in HepG2 cells on collagen-coated cover slips in a four-well plate as described above. Infected cultures were fixed at 12, 24, 48, 60, and 65 hpi with 4% PFA. The 12, 36, and 48 h EEFs were stained with anti-CSP mouse monoclonal (3D11) and rabbit anti-UIS4 antibody that stained the EEF and PVM, respectively. The immunoreactivity was revealed with Alexa Fluor 488-conjugated goat anti-mouse and Alexa Fluor 594-conjugated chicken anti-rabbit secondary antibodies at 1:1,000 dilution, respectively. The 60 and 65 h EEFs were stained with MSP1 mouse monoclonal and rabbit anti-UIS4 antibody that stained the hepatic merozoites and PVM, respectively. To reveal the immunoreactivity, Alexa Fluor 488-conjugated goat anti-mouse secondary antibody and Alexa Fluor 594-conjugated chicken anti-rabbit secondary antibody at 1:1,000 dilution were used. Nuclei were stained with DAPI, and coverslips were mounted using ProLong Gold Anti-Fade Reagent and sealed with nail polish. The slide was visualized under a Nikon Eclipse upright fluorescence microscope, and images were captured using the NIS-Elements AR software.

To analyze the fate of anti-HA or 3D11 antibody-treated *Pb*RON6::3xHA transgenic sporozoites in the mouse macrophage (RAW) cell line, the cultures were fixed at 7 hpi. The sporozoites were stained with 3D11 (1:1,000 dilution), and macrophage endosomes were stained with rabbit anti-Rab5 antibody (1:100 dilution). Primary antibodies were removed after 1 h incubation, washed three times with PBS followed by incubation with Alexa Fluor 488-conjugated goat anti-mouse secondary antibody and Alexa Fluor 594-conjugated chicken anti-rabbit secondary antibody at 1:1,000 dilution. Nuclei were stained with DAPI. Coverslips were mounted using ProLong Gold Anti-Fade Reagent and sealed with nail polish. The slide was visualized under a Nikon Eclipse upright fluorescence microscope, and images were captured using the NIS-Elements AR software. The number of antibody-treated *Pb*RON6::3xHA transgenic sporozoites present inside and outside the RAW cells was quantified.

To test the antisera raised against 110–281 AAs of PbRON6, an indirect IFA was performed using schizonts and free merozoites of the *Pbron6::3xHA* line. The schizont and free merozoites were fixed with 4% PFA, blocked with 3% BSA solution, and stained with anti-HA rabbit monoclonal antibody at 1:100 dilution and mouse antisera specific for rPbRON6^110–281^ at 1:100 dilution. The immunoreactivity was revealed using Alexa Fluor 488-conjugated goat anti-mouse secondary antibody and Alexa Fluor 594-conjugated chicken anti-rabbit secondary antibody at 1:1,000 dilution. Nuclei were stained with DAPI. To test the immunoreactivity of antisera raised against 110–281 AAs, sporozoites were fixed with 4% PFA, and staining was done under non-permeabilized and permeabilized conditions as mentioned above. Anti-SIMP antibody was used as a sporozoite-specific marker.

### Quantification of *Pb*RON6::3xHA levels in sporozoite membrane

To determine the levels of *Pb*RON6 protein in sporozoite membrane, 3,000 *freshly dissected Pb*RON6::3xHA transgenic sporozoites were added per well on a spotted slide (Fisher Scientific, Cat #9991090) and immediately fixed at room temperature, or alternatively, sporozoites were maintained at 37°C for 30 min in the presence or absence of HepG2 cells in a Nunc Lab-Tek II Chamber Slide (Thermo Scientific, Cat #154453). After incubation, cells were fixed with 4% PFA, followed by staining with 1:1,000 dilution of 3D11 and 1:100 dilution of anti-HA rabbit monoclonal antibody (Abcam, Cat #AB18181). The immunoreactivity of 3D11 and anti-HA antibody was revealed with Alexa Fluor 488-conjugated goat anti-mouse and Alexa Fluor 594-conjugated chicken anti-rabbit secondary antibodies used at 1:1,000 dilution, respectively. The sporozoites were visualized under a Nikon Eclipse upright fluorescent microscope. The intensity of 3xHA on the sporozoite membrane was quantified by using NIS Elements AR software.

### Analysis of *in vivo* sporozoite infectivity of *PbΔron6*

To determine the *in vivo* infectivity of *PbΔron6* sporozoites, two groups containing 6 and 7 C57BL/6 mice were exposed to either 5 × 10^3^ or 1 × 10^4^ mutant or WT sporozoites, by an intravenous route. The sporozoite infectivity was monitored by the Giemsa-stained tail blood smears for determining pre-patency, which is defined as the time required for the appearance of blood stage infection in mice following sporozoite exposure. A delay in pre-patency compared to WT was considered as reduced infectivity. Spleens were collected prior to terminally euthanizing the animals. Length and weight of the isolated spleens were measured, and the samples were used for histopathological analysis.

### Sporozoite inside-out assay

To investigate the efficiency of hepatocyte invasion, 2 × 10^4^ *PbΔron6* or WT sporozoites were added to HepG2 monolayers cultured on collagen (Corning, Cat #354236) coated coverslips, in a four-well plate. The cultures were incubated for 1 h at 37°C in a CO_2_ incubator, followed by fixing with 4% PFA. Sporozoite invasion was assessed by a dual staining method as described previously (48). Briefly, following non-specific blocking with 3% BSA, the extracellular sporozoites were first stained under non-permeabilized conditions with anti-CSP (3D11) monoclonal antibody, and immunoreactivity was revealed with anti-mouse Alexa Fluor 488-conjugated goat secondary antibody. In the next step, the cultures were permeabilized with a 1:3 ratio of cold acetone-methanol solution, followed by non-specific blocking. The permeabilized cultures were stained for a second time with 3D11 monoclonal antibody, and immunoreactivity was revealed using Alexa Fluor 594-conjugated goat anti-mouse secondary antibody (Thermo Scientific, Cat #A11032). Nuclei were stained with DAPI. The slide was mounted with a coverslip using ProLong Gold Anti-Fade Reagent and sealed with nail polish. The slide was visualized under a Nikon Eclipse upright fluorescent microscope, and images were captured and analyzed using the NIS-Elements AR software. The number of green and red sporozoites was scored from 20 random fields, which represented the extracellular and total sporozoites, respectively. The percentage of sporozoite invasion was assessed by the following formula:

Percentage invasion = ([Red parasites − Green parasites]/Red parasites) × 100

### Exocytosis assay for *Pb*RON6

To assess whether *Pb*RON6 is secreted by sporozoites during exocytosis, approximately 5 × 10^4^ *Pbron6*::3xHA transgenic sporozoites adjusted to 20 µL of complete RPMI medium were incubated at 37°C for either 30 or 60 min. The sporozoite samples were centrifuged at 10,000 × *g* for 10 min at 4°C. Supernatants and pellets were collected separately, and the samples were denatured with Laemmli Sample Buffer, resolved on a 12% SDS-PAGE gel, and immunoblotted. CSP and RON6 levels were analyzed in the supernatant and the pellet fractions by probing with 3D11 and anti-HA rabbit polyclonal antibodies, respectively. The immunoreactivity was revealed using HRP-conjugated horse anti-mouse (Cell Signaling Technology, Cat #7076) and goat anti-rabbit secondary antibodies (Cell Signaling Technology, Cat #7074) in the presence of chemiluminescence reagent (G-Biosciences, ECL #786-003), and visualized using ChemiDoc Imaging System (Bio-Rad).

To analyze the sporozoite precipitin reaction in the presence of antibodies, 5 × 10^3^ *Pbron6::3xHA* transgenic sporozoites were incubated with either 3D11 or anti-HA rabbit monoclonal antibody for 30 min at 37°C. After incubation, the sporozoite suspension was placed on a microscope glass slide (Corning, Cat #2948) and visualized under a Nikon Eclipse upright fluorescent microscope, and images were captured using the NIS-Elements AR software.

### Sporozoite *in vivo* invasion efficiency and estimation of parasite burden in the liver

Two groups of C57BL/6 mice (*n* = 9/group) were intravenously infected with 5 × 10^3^ sporozoites of *PbΔron6* or WT *P. berghei*. Three animals from each group were sacrificed, and livers were isolated at 6, 12, and 48 hpi. Total RNA was isolated from infected livers using the guanidinium thiocyanate-phenol-chloroform extraction method (74). cDNA was generated from 2 µg of total RNA from each liver sample. qRT-PCR was performed, and the Ct values were determined for *P. berghei 18S rRNA* for livers isolated at 6 and 12 h and *msp1* for the livers isolated at 48 hpi. Mouse *gapdh* was used to normalize the Ct values of parasite genes following 2^−ΔΔC*t*^ method. Table S1 shows the details of primers used for qRT-PCR.

### RBC invasion assay

To quantify merozoite invasion of RBC, *in vitro* cultures of *PbΔron6* mutant and WT *P. berghei* asexual stages were set up, and schizonts were enriched by density gradient centrifugation using Histodenz (Sigma, Cat #D2158). Twenty-five milliliters of overnight *in vitro* schizont culture was topped on 10 mL of 60% histodenz solution and centrifuged at 380 × *g* for 20 min without a break. The brown interphase of the schizonts was collected and washed two times with culture medium. The purified schizonts were quantified by using a hemocytometer, and nearly 10^9^ parasites of WT or *PbΔron6* were injected into two groups of Swiss Albino mice (*n* = 5/group). To capture the early snapshots of merozoite invasion and propagation, blood smears were done at 6-h intervals till 48 h and parasitemia was determined by Giemsa staining.

### Generation of antisera against *Pb*RON6^110–281^

To generate antisera against *Pb*RON6, nucleotide sequence encoding AAs 110–281 of *Pbron6* was PCR amplified using the primers P35/P36 (See Table S1 for primer details) and cloned into EcoRI and HindIII restriction sites of pET21a vector (Millipore-Sigma, Cat #69740). The nucleotide sequence of *ron6* in the plasmid was confirmed by Sanger sequencing and was transformed into Rosetta 2(DE3) competent cells (Millipore-Sigma, Cat #71397). Recombinant *Pb*RON6 (r*Pb*RON6) was induced with 0.7 mM IPTG at 37°C. Protein was affinity purified using Ni-NTA agarose beads (G Biosciences, Cat #786-1547) and eluted at 10 mM imidazole. Purified r*Pb*RON6 was concentrated by dialysis, and the protein purity was assessed by 15% SDS-PAGE.

Seven female BALB/c mice (5–7 weeks old) were immunized with rPbRON6 with Freund’s complete adjuvant (Sigma, Cat #F5881). Ten days post-priming, four booster doses were given at every 14-day interval with r*Pb*RON6 in Freund’s incomplete adjuvant (Sigma, Cat #F5506). Immune sera were collected 2 weeks after the final booster dose. The immune sera were used to perform an indirect IFA.

### IP and LC/MS analysis

IP was performed using the Pierce crosslink IP kit (Thermo Scientific, Cat #26147) as per the manufacturer’s instructions. Briefly, *Pb*RON6::3xHA transgenic schizonts were cultured overnight *in vitro*. Schizonts were purified using a 60% Histodenz gradient as described earlier. Parasites were lysed with 0.15% saponin (Sigma, Cat #47036), and the sample for immuno-precipitation was prepared in IP lysis buffer. Rabbit monoclonal anti-HA antibody was cross-linked to protein A/G plus agarose beads and subsequently incubated with parasite lysate (>1 mg) overnight at 4°C. After overnight incubation, the protein A/G beads were washed with IP lysis buffer, and the proteins bound to the beads were eluted in elution buffer (Tris-glycine, pH 2.8) provided with the kit. Rabbit IgG was used as a control for pulldown. The successful immune precipitation was confirmed by Western blot using anti-HA rabbit monoclonal antibody.

For LC/MS analysis, the proteins present in the elution fraction after IP were digested by in-solution trypsin digestion. The samples were reduced using 20 mM DTT followed by treatment with 40 mM iodoacetamide at room temperature in the dark. For in-solution trypsin digestion, a 1:50 (wt/wt) trypsin to protein ratio was used. Proteins in each elution sample were digested by trypsin (New England Biolabs, Cat #P8101S) and incubated at 37°C for 16 h with shaking. After trypsin digestion, the sample was acidified with formic acid to a final concentration of 0.1% (Thermo Scientific, Cat #28905). The acidified peptides were desalted using Pierce C-18 spin column (Thermo Scientific, Cat #89870). Briefly, the C18 columns were activated with 1 mL of 2× acetonitrile (ACN) (Merck, Cat #AX0156) prepared in LC-MS grade water. The columns were equilibrated in 0.5% trifluoroacetic acid (TFA) (Merck, Cat #80457) and 0.5% acetonitrile solution. The acidified peptides were bound to the equilibrated columns and washed twice with 1 mL of 0.5% ACN and 0.5% TFA solution. The peptide elution was done in 50 µL of elution buffer (70% ACN and 0.1% TFA) in LC-MS grade water (Thermo Scientific, Cat #W6212). The eluted peptide samples were dried in a SpeedVac vacuum concentrator and stored at −80^ο^C till further use.

The LC/MS analysis was executed on an Orbitrap Q-Exactive HF mass spectrometer, coupled with a Nano LC 1000 instrument (Thermo Fisher Scientific, USA), supported with Thermo Scientific Xcalibur software. The eluted peptides were reconstituted with 0.1% formic acid, and approximately 2 µg of peptide sample was resolved on a PepMap RSLC C18 column (2 µm particle size, 100 Å pore size, and 75 µm × 50 cm). The raw data files from Orbitrap Q-Exactive HF were acquired on Proteome Discoverer, version 2.2, using the SEQUEST algorithm. The predicted peptides were searched against the UniProt database of *P. berghei*. The data were normalized with peptides detected in control IgG IP. The GO term analysis was carried out via PlasmoDB GO term analysis tool. The data were clustered according to the fold enrichment, and the number of proteins involved in a particular pathway was detected in IP/MS results.

### Tissue sectioning for analysis of histopathological changes

The isolated spleen tissues were frozen, and optimal cutting temperature compound (OCT compound, Scigen, Cat #23-730-625) was used to embed tissue samples prior to sectioning on a cryostat. The block was incubated at −80°C for 2–3 h, and the cryostat (LEICA CM 1850 UV-1-1) temperature was adjusted to −20°C. After the 3-hour incubation, the block was placed on the chuck associated with the rotatory microtome, and 2 mm thick sections were sliced and transferred onto positively charged microscopy slides (PathnSitu Biotechnologies, Cat #PS-011-72).

### Hematoxylin and eosin staining (H&E staining)

Prior to H&E staining, the sections were deparaffinized by immersing the slide in xylene solution (Sigma, Cat #296333) for 5 min. The sections were dehydrated sequentially using 100%, 90%, 70%, 50%, and 30% alcohol, each for 10 min, followed by an additional wash with double distilled water for 5 min. The tissue sections were incubated with hematoxylin (Sigma, Cat #H9627) for 20 min. The sections were washed with double-distilled water for 10 min followed by treatment with 1% eosin (Sigma, Cat #E4009) for 5 min, and finally washed with double-distilled water for 2 min. Sections were rehydrated sequentially using 30%, 50%, 70%, and 90% alcohol for 5 min each, and cleared with xylene for 5 min. The sections were dried by gently pressing on filter paper and mounted with DPX (Sigma, Cat #100579).

### Flow cytometry of splenocytes

Spleens were isolated from WT infected, *PbΔron6* infected, and age-matched uninfected C57BL/6 mice (*n* = 5 per group). Splenocyte suspensions were obtained by crushing the spleens between two clean glass slides, and the suspension was passed through a 70 µm cell strainer (SPL Lifesciences, Cat #93070). The single-cell suspensions were centrifuged at 1,500  rpm for 3 min at 4°C. The pellets were washed with sterile PBS. A hemocytometer was used to observe cell viability following trypan blue staining, and viable cells were quantified. For flow cytometry analysis, 2 × 10⁶ cells were resuspended in 100 µL FACS buffer (BD Biosciences, Cat #554657) in a 1.5 mL microcentrifuge tube and stained with 1 µL each of anti-mouse CD3-FITC (BD Biosciences, Cat #553081) and anti-mouse CD20-PE (BD Biosciences, Cat #567250). Samples were incubated for 45 mins on ice and centrifuged at 1,500 rpm for 3 min at 4°C. The cells were then washed with PBS, centrifuged again, and the pellet was resuspended in 200 µL FACS buffer for analysis. The lymphocyte data were acquired by flow cytometry (LSR Fortessa, BD Biosciences, USA), and the cells were gated broadly on FSC-A Vs SSC-A and analyzed using FlowJo software (https://www.flowjo.com, FlowJo Version 10.8.1).

### Statistical analysis

GraphPad Prism v9.0 and above was used for statistical analysis. Normality was tested for all data using the Shapiro-Wilk test and Anderson-Darling test before analysis. Statistical analyses were performed using Mantel-Cox tests for survival analysis, Mann-Whitney test for extracellular sporozoite counting in Raw cell line, for measuring EEF area at 36 and 65 h post-infection, for measuring number of hepatic merozoites at 60 h post-infection, and for determining percentage frequency of larger EEFs at 65 h post infection. An unpaired *t*-test was used to calculate the statistical differences for the sporozoite inside-out assay and for the quantification of *Pb18S rRNA* and *MSP1* from infected mice livers. One-way ANOVA with Tukey’s multiple comparison test was performed to determine the significance for blood stage propagation of WT and *PbΔron6* mutant parasites in mice, for quantification of midgut and SG sporozoites and also for the assessment of spleen weights from mice infected with WT and *PbΔron6* mutant sporozoites, and Dunnett’s multiple comparison test for blood stage propagation of WT and *Pbron6* complemented parasites in mice. Kruskal-Wallis test with Dunn’s multiple comparisons test was used for the assessment of spleen lengths from mice infected with WT and *PbΔron6* mutant sporozoites. One-way ANOVA with Holm-Šídák’s multiple comparisons test was performed to determine statistical significance for oocyst burden in mosquitoes.

## SUPPLEMENTAL MATERIAL

Supplemental figures and table mbio.01941-25-s0001.pdf Fig. S1 to S5 and Table S1.

ASM does not own the copyrights to Supplemental Material that may be linked to, or accessed through, an article. The authors have granted ASM a non-exclusive, world-wide license to publish the Supplemental Material files. Please contact the corresponding author directly for reuse.
