# Supplementary material for: Plasmodium berghei rhoptry neck protein 6 maintains parasite infectivity and virulence
Source: mBio. 2025 Sep 25;16(11):e01941-25. doi: 10.1128/mbio.01941-25 (PMC12607912; doi:10.1128/mbio.01941-25)
Supplement: Supplemental figures and table — Fig. S1 to S5 and Table S1. [file mbio.01941-25-s0001.pdf]

Fig. S1

A

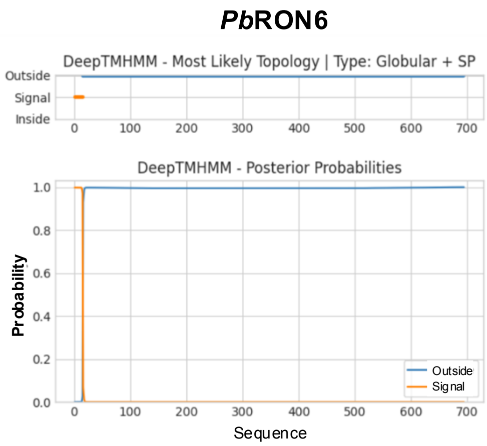

- Length of the protein: 694 aa
- Nature: Globular protein
- No transmembrane domain
- Signal peptide: 1-15
- Extracellular domain: 16-694

B

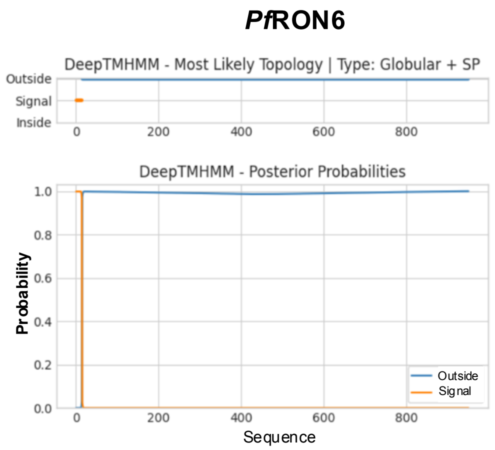

- Length of the protein: 950 aa
- Nature: Globular protein
- No transmembrane domain
- Signal peptide: 1-15
- Extracellular domain: 16-950

C

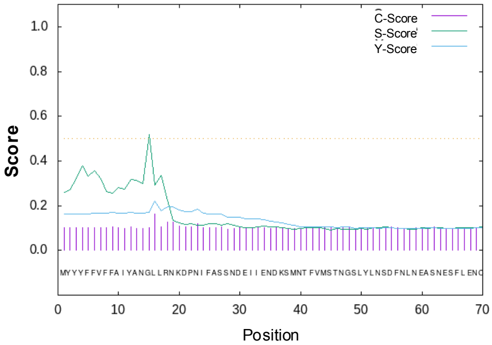

# Measure	Position	Value	Cutoff	signal peptide?
Max. C	16	0.162		
Max. Y	16	0.220		
Max. S	15	0.517		
Mean S	1-15	0.317		
D	1-15	0.272	0.450	NO

D

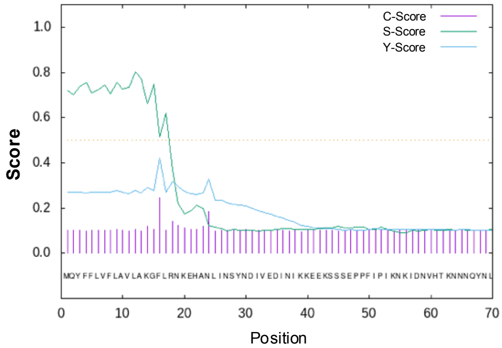

# Measure	Position	Value	Cutoff	signal peptide?
Max. C	16	0.245		
Max. Y	16	0.423		
Max. S	12	0.802		
Mean S	1-15	0.732		
D	1-15	0.590	0.450	YES

E F

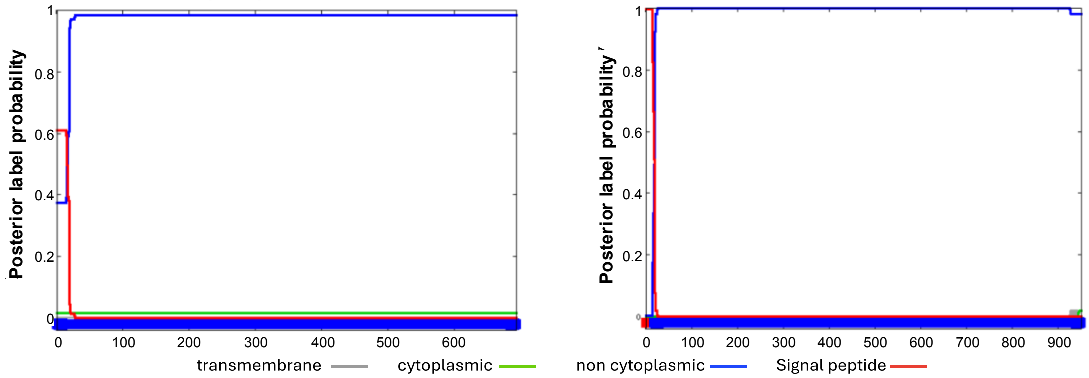

G

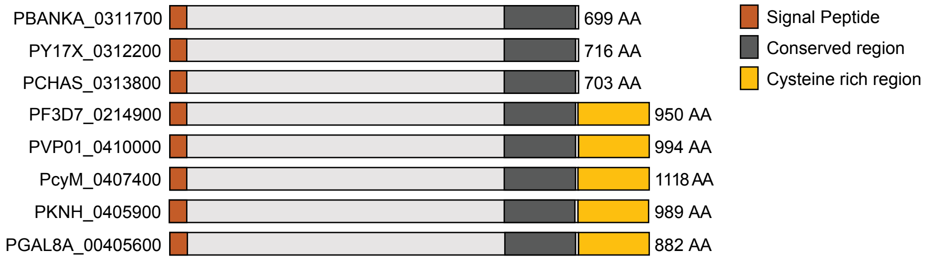

Fig S1: Prediction of signal peptide (SP) and probable cellular location of *P. berghei* and *P. falciparum* RON6 sequence using different computational tools. (A and B) Deep TMHMM posterior probability prediction supporting a globular nature of *P. berghei* and *P. falciparum* RON6, (C and D) Prediction of signal peptide in *P. berghei* and *P. falciparum* RON6 using Signal P 4.1. (E and F) Posterior label probability prediction of cellular location of *P. berghei* and *P. falciparum* RON6 using Phobious web server. (G) Linear models of RON6 orthologues from different species of *Plasmodium*. Signal peptide, conserved region, and cysteine-rich domain are highlighted respectively in brown, grey, and yellow colour.

Fig. S2

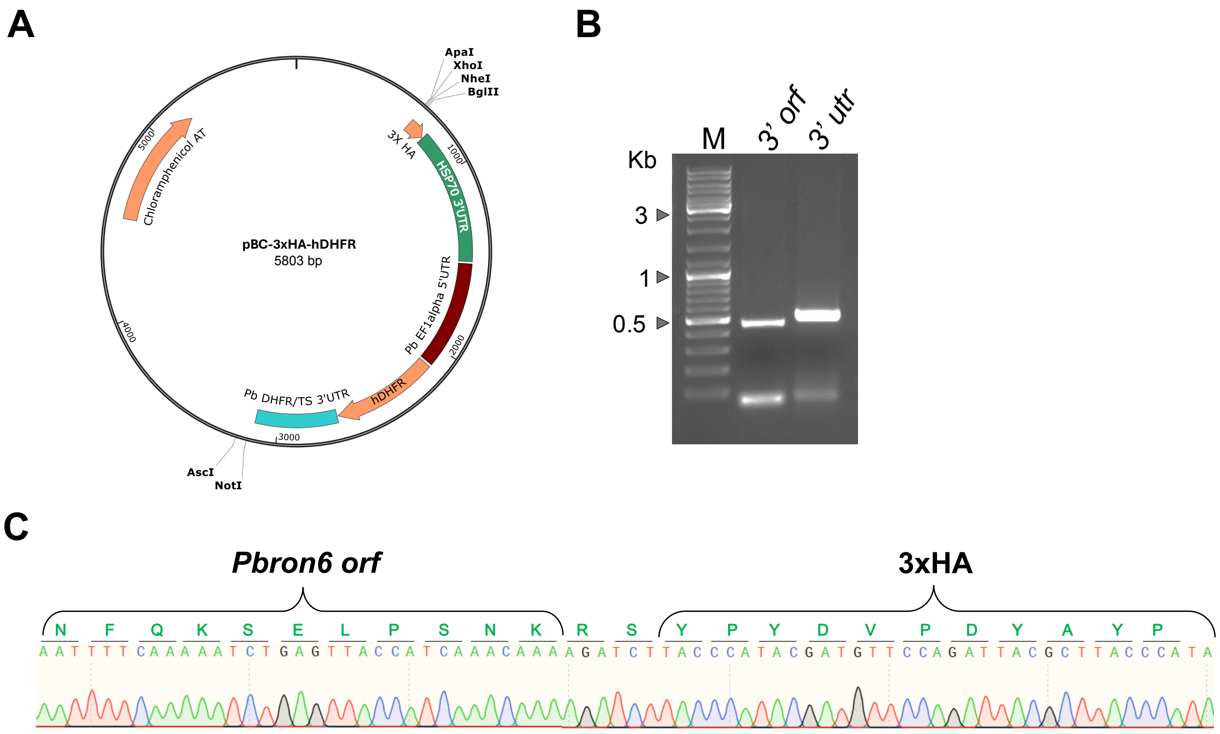

Fig S2: (A) Schematic of pBC-3xHA-hDHFR plasmid map used for generating *Pbron6::3xHA* transgenic line. The plasmid carries a 3xHA tag, *PbHSP70 3'UTR* and a hDHFR cassette, flanked by *Pbef1α 5'UTR* and *PbDHFR/TS 3'UTR*. A chloramphenicol antibiotic resistance marker aids in bacterial selection. (B) Agarose gel showing the amplification of *Pbron6 3' orf* and *3' utr*. (C) Sanger sequencing chromatogram of recombined *Pbron6* locus showing *3xHA* tag in-frame with *Pbron6 orf*.

Fig. S3

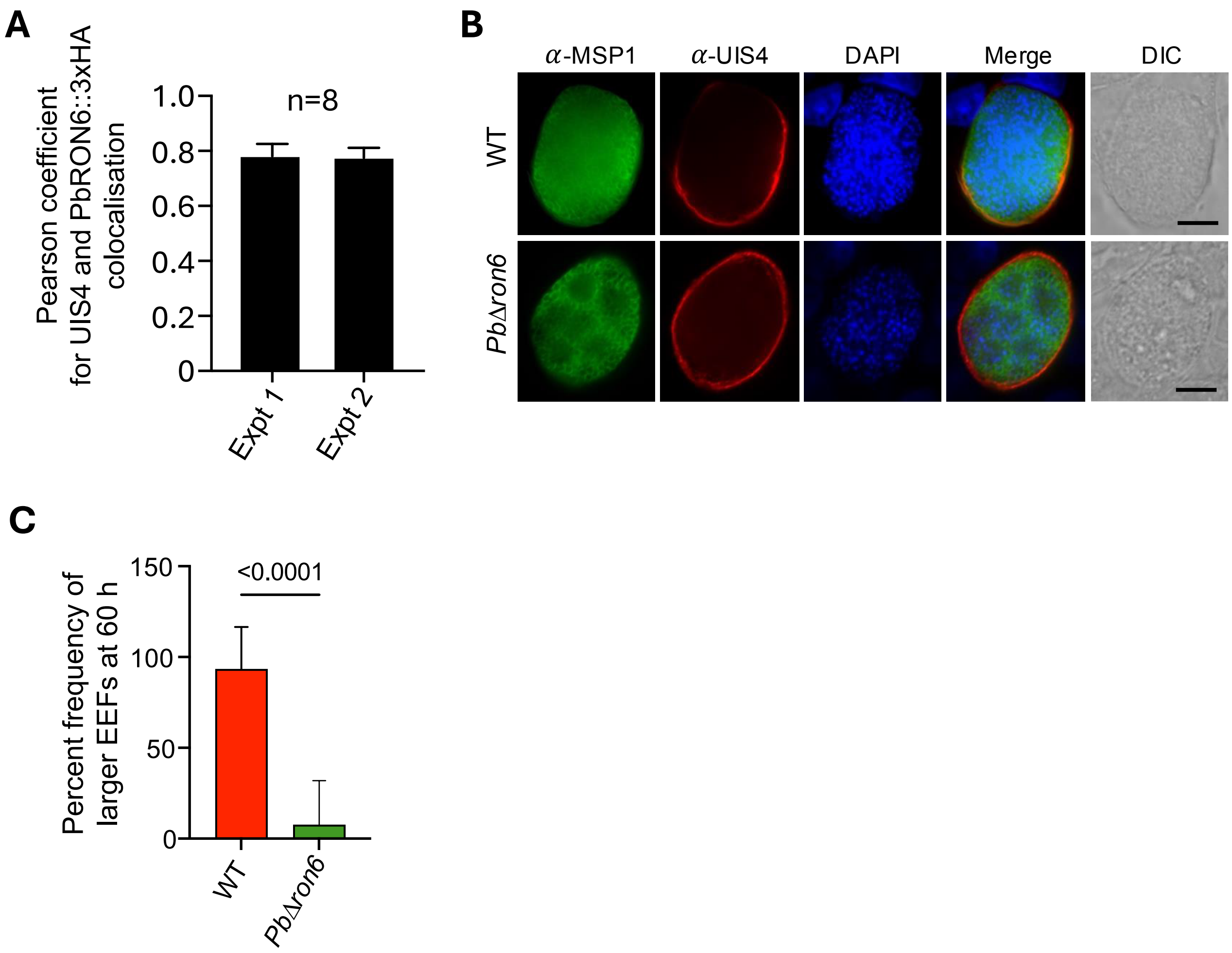

Fig S3: (A) Bar graph showing the Pearson's coefficient for colocalization of UIS4 and PbRON6::3xHA in *in vitro* EEFs at 36 h. Data was collected from two independent experiments (n=8 per experiment). (B) EEFs at 60 h of WT (top panel) and PbΔron6 (lower panel). Very few of the mutant EEFs were comparable in size to WT, but the cytomere size was small and hepatic merozoites were less. (B) The graph corresponds to the percentage frequency of PbΔron6 at 60 h EEF, whose size was comparable to WT. Number of fields viewed, n=20. Error bars represent mean with standard deviation. Statistical difference was determined by Mann-Whitney test.

Fig. S4

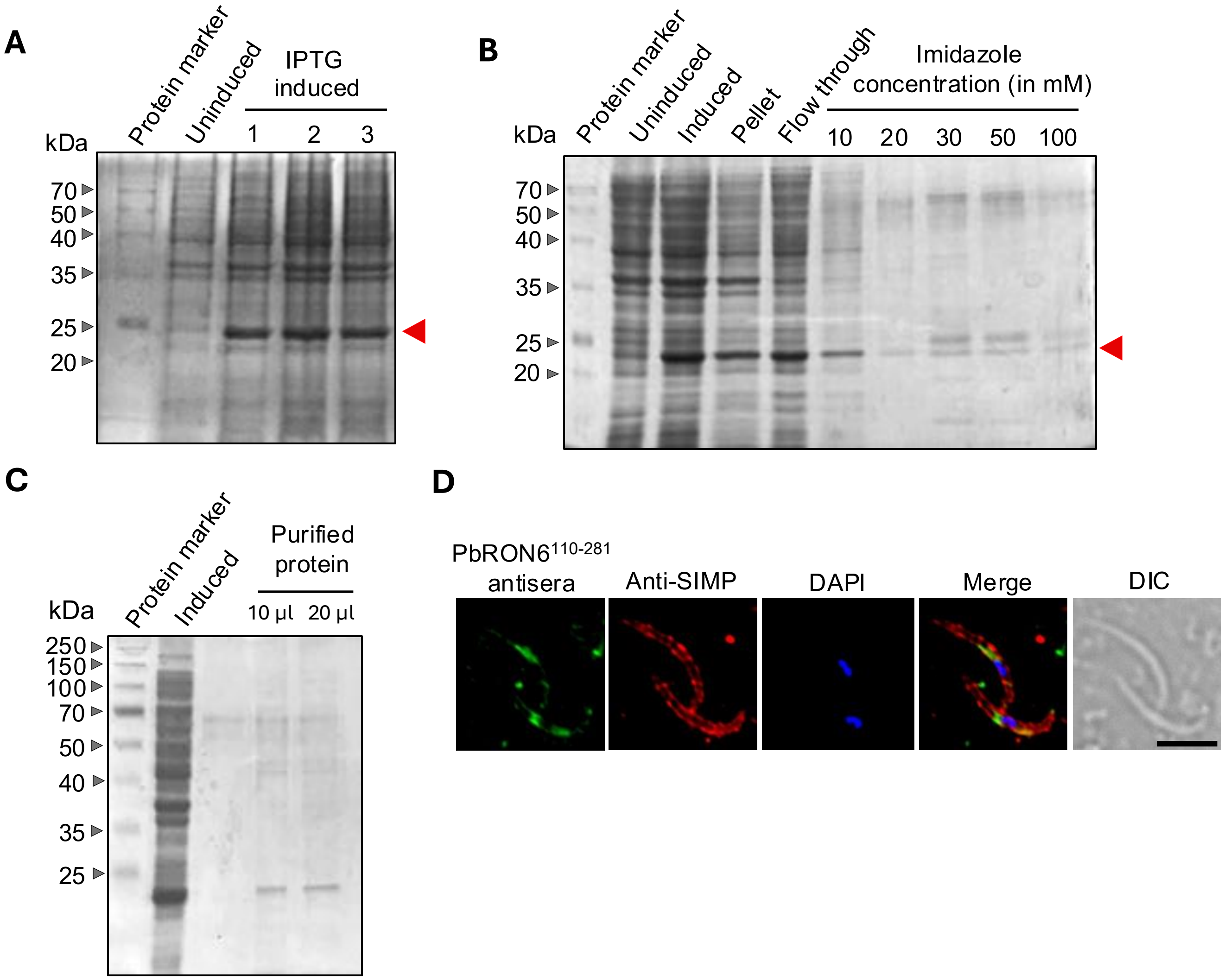

Fig S4: Recombinant protein expression, purification and generation of mouse polyclonal antisera against PbRON6 110-281 amino acids (rPbRON6$^{110\text{-}281}$). (A) SDS-PAGE gel showing lysates of Rosetta DE3, uninduced or induced with 0.7 mM IPTG at 37°C for 20 h. Red arrows showing induction of rPbRON6$^{110\text{-}281}$ from three independent colonies (1, 2 and 3). (B) SDS-PAGE gel showing the purification profile of rPbRON6$^{110\text{-}281}$ (red arrows) from bacterial cultures. For elution of rPbRON6$^{110\text{-}281}$ bound to Ni-NTA resin, an imidazole concentration of 10-100 mM was used and maximal recombinant protein elution was noted at 10 mM concentration. (C) SDS-PAGE gel showing analysis of purified protein fractions. (D) Immunofluorescence analysis of sporozoites with PbRON6$^{110\text{-}281}$ mouse antisera and anti-rabbit SIMP antisera. SIMP antibody was used as a sporozoite membrane marker. The immunoreactivity was revealed using anti-mouse AlexaFluor 488 and anti-rabbit AlexaFluor 594 secondary antibodies. DAPI was used to stain the nuclei. Scale bar - 5 µm.

Fig. S5

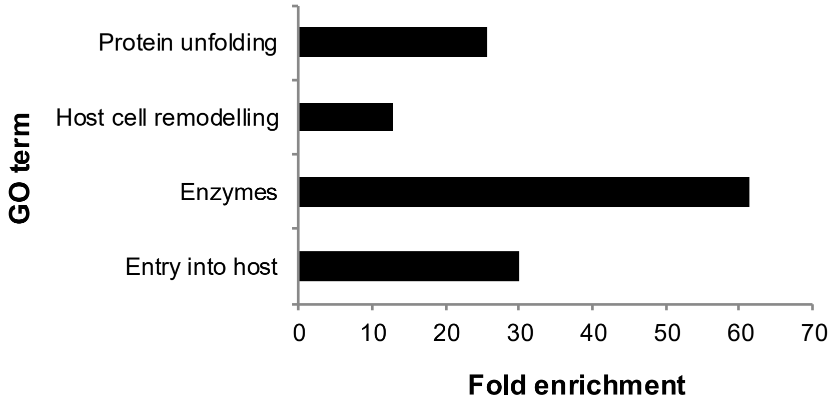

Fig S5: Validation of GO term ID for each cluster with fold enrichment >10 and $p<0.05$ identified in co-immunoprecipitation of PbRON6::3xHA schizont lysate using rabbit anti-HA monoclonal antibody.

Table S1: List of primers

Primers used to generate *Pbron6* targeting constructs

DNA Construct	Primer ID	Sequence	Enzymes	Product (bp)	Primer description
Pbron6::3xHA construct	P1	GTGCTCGAGCTAATTGTATCATGCTCAATCTG	XhoI	455	*Pbron6 orf* forward
	P2	CCAAGATCTTTTGTTTGATGGTAACTCAGATT	BglII		*Pbron6 orf* reverse
	P3	GGTGCGGCCGCGAAAAACAATGAGGTATGACCA	NotI	507	*Pbron6 3' utr* forward
	P4	CACGGCGCGCCTCCCTCTTATACTTTATCGCCA	AscI		*Pbron6 3' utr* reverse
Pbron6-KO construct	P5	GTGCTCGAGTTTCTATTATTTGCATTTATTCCCTTTGC	XhoI	784	*Pbron6 5' utr* forward
	P6	CCAAAGCTTTTTTGAGAGTAAACACACTTAAC	HindIII		*Pbron6 5' utr* reverse
	P3	GGTGCGGCCGCGAAAAACAATGAGGTATGACCA	NotI	800	*Pbron6 3'utr* forward
	P7	CACGGCGCGCCTTCAAAAGAGTATATTGATTATGTT	AscI		*Pbron6 3'utr* reverse
Pbron6 complementation construct	P8	CCAAAGCTTATGTATTATTATTTTTTTGTTTTTT	HindIII	2596	*Pbron6 orf* (full length) forward
	P9	GGTGCGGCCGCTTATTTGTTTGATGGTAACTCAG	NotI		*Pbron6 orf* (full length) reverse

Primers used for *Pbron6* int PCRs

Parasite line	Primer ID	Sequence	Product (bp)	Primer description
Pbron6::3xHA integration Confirmations	P10	AATTCCATCTATATGTTTGAAGAT	798	*Pbron6::3xHA 5'* int forward
	P11	ATATACAACAAAAAGGAGGTACAC		*Pbhsp70 3' utr* reverse
	P12	TAAACACAAATGATGTTTTTTTCCTTC	618	*Pbdhfr 3' utr* forward
	P13	CTGCTGCAAAAAAATTGACGGC		*Pbron6 3'* int reverse
Pbron6-KO integration confirmation	P14	TATTTTCATGATATTTTCATAATGTT	1631	*Pbron6 5'* int forward
	P15	CAGGTCTTCTTACCCATAATCACC		*Pbef1α* promoter reverse
	P16	GATTTTTAAAATGTTTATAATATGATT	1256	*Pbdhfr 3' utr forward*
	P17	TACAATGGATGGTCCTATGGC		*Pbron6 3'* int reverse
	P18	GTTTATAAGGAAGCCATGAATC	502	Selectable marker forward
	P19	GCGTCGTATACAAAGTGGTAT		Selectable marker reverse
	P20	ATGGCAACAAAATAAATAGTCAAG	516	*Pbron6 orf* confirmation forward
	P21	CGTCTTCCTTATCTTCGTCAGA		*Pbron6 orf* confirmation reverse

Pbron6 complementation integration Confirmation	P20	ATGGCAACAAAATAAATAGTCAAG	516	*Pbron6 orf* confirmation forward
	P21	CGTCTTCCTTATCTTCGTCAGA		*Pbron6 orf* confirmation reverse
	P14	TATTTTCATGATATTTTCATAATGTT	1642	*Pbron6 5'* int forward
	P22	CGTTATATCCATCTTCGACAGA		*Pbron6 orf* int reverse
	P23	CATGTTTACTATCGACAAAAAAAT	1607	*Pbron6 orf* int forward
	P17	TACAATGGATGGTCCTATGGC		*Pbron6 3'* int reverse
	P18	GTTTATAAGGAAGCCATGAATC	502	Selectable marker forward
	P19	GCGTCGTATACAAAGTGGTAT		Selectable marker reverse

Primers used for the confirmation of *Pbron6::3xHA* in frame fusion

Parasite line	Primer ID	Sequence	Product (bp)	Primer description
Pbron6::3xHA	P24	CCCTTCAAAAATATATGAATGAAACAGG	422	*Pbron6::3xHA* forward
	P11	ATATACAACAAAAAGGAGGTACAC		*Pbhsp70 3'utr* reverse

Primers used for qRT-PCRs

Gene Name	Primer ID	Sequence	Product (bp)	Primer description
*Pbron*6 qRT PCR	P25	TAAAGGCGCTGATGAAATGGA	136	*Pbron6* qPCR forward
	P26	GTCTTCCTTATCTTCGTCAGA		*Pbron6* qPCR reverse
Pb18S rRNA qRT PCR	P27	GGAGATTGGTTTTGACGTTTATGT	134	*Pb18S rRNA* qPCR forward
	P28	AAGCATTAAATAAAGCGAATACATCCTTAC		*Pb18S rRNA* qPCR reverse
Pbmsp1 qRT PCR	P29	AATGCTGGATGTTTTAGATATGA	129	*Pbmsp1* qPCR forward
	P30	ATCACATCCACCATTGTTGTTTCC		*Pbmsp1* qPCR reverse
Mouse *Gapdh* qRT PCR	P31	CCTCAACTACATGGTCTACAT	122	Mouse *Gapdh* qPCR forward
	P32	GCTCCTGGAAGATGGTGATG		Mouse *Gapdh* qPCR forward
Pbef1α qRT PCR	P33	TGGAACCACCCAAAAGACCA	154	*Pbef1α* qPCR forward
	P34	ACAACAGCAGATGGAGCGAA		*Pbef1α* qPCR reverse

Primers used for recombinant protein expression

DNA Construct	Primer ID	Sequence	Enzymes	Product (bp)	Primer description
Pbron6 recombinant protein	P35	GCGAATTCATGGCAACAAAATAAATAGTCAAG	EcoRI	517	*Pbron6* recombinant forward
	P36	CCGAAGCTTCGTCTTCCTTATCTTCGTCAG	HindIII		*Pbron6* recombinant forward

orf - open reading frame, utr - untranslated region and int - integration
